# Optimisation of the Anti-*Trypanosoma brucei* Activity of the Opioid Agonist U50488

**DOI:** 10.1002/cmdc.201100278

**Published:** 2011-08-10

**Authors:** Victoria C Smith, Laura A T Cleghorn, Andrew Woodland, Daniel Spinks, Irene Hallyburton, Iain T Collie, N Yi Mok, Suzanne Norval, Ruth Brenk, Alan H Fairlamb, Julie A Frearson, Kevin D Read, Ian H Gilbert, Paul G Wyatt

**Affiliations:** Drug Discovery Unit, College of Life Sciences, James Black Centre, University of DundeeDundee, DD1 5EH, Scotland (UK)

**Keywords:** antiprotozoal agents, human African trypanosomiasis, medicinal chemistry, U50488

## Abstract

Screening of the Sigma–Aldrich Library of Pharmacologically Active Compounds (LOPAC) against cultured *Trypanosoma brucei*, the causative agent of African sleeping sickness, resulted in the identification of a number of compounds with selective antiproliferative activity over mammalian cells. These included (+)-(1*R*,2*R*)-U50488, a weak opioid agonist with an EC_50_ value of 59 nm as determined in our *T. brucei* in vitro assay reported previously. This paper describes the modification of key structural elements of U50488 to investigate structure–activity relationships (SAR) and to optimise the antiproliferative activity and pharmacokinetic properties of this compound.

## Introduction

Human African trypanosomiasis (HAT), or African sleeping sickness, is a parasitic disease caused by protozoa parasites of the species *Trypanosoma brucei* and is transmitted by the tsetse fly. HAT is endemic in certain regions of sub-Saharan Africa, threatening about 60 million people in 36 countries. The disease is a major cause of morbidity and mortality in these developing regions, resulting in an estimated 30 000 deaths each year.[1]

Phase 1 of the disease, after a bite by an infected fly, results in a systemic infection from the extracellular spread of parasites, causing episodes of fever, headache, sweating, and swelling of the lymph nodes. Phase 2 of the disease results from the spread of infection into the central nervous system. The term *sleeping sickness* derives from the symptoms of this second phase, in which the circadian rhythm is disturbed, resulting in bouts of fatigue alternating with manic periods, progressing to daytime slumber and nighttime insomnia. Without treatment, the disease is fatal; progressive mental deterioration leads to coma and death. The current therapeutic product profile for HAT requires new clinical candidates to show activity in models of phase 2 of the disease, therefore requiring compounds to cross the blood–brain barrier.[2]

Despite the burden of HAT and other neglected diseases, there is a lack of validated drug discovery targets and lead compounds for these diseases.[1, 3] To address this gap, a number of approaches to generate hits have been taken: the exploitation of parasite-specific targets with little history of drug discovery, the exploitation of target families with a history of drug discovery for other indications, and hit identification through phenotypic (in vitro whole-parasite) screening. The Drug Discovery Unit (DDU) at the University of Dundee (http://www.drugdiscovery.dundee.ac.uk/) is taking each of these approaches to develop a portfolio of projects to discover drugs for HAT. Herein we report the optimisation of (+)-(1*R*,2*R*)-U50488 (**1**), a hit compound discovered by screening the Sigma–Aldrich Library of Pharmacologically Active Compounds (LOPAC) against *T. brucei* in culture, using MRC-5 cells as a mammalian cell line counter-screen to exclude nonselective compounds. We recently reported the output of this screen,[4] and herein we discuss the medicinal chemistry programme around one of the hits, (+)-(1*R*,2*R*)-U50488 (**1**).

Compound **1** (Table 1), is the enantiomer of (−)-(1*S*,2*S*)-U50488 (**2**), which is a potent agonist of the κ-opioid receptor. In contrast, (+)-(1*R*,2*R*)-U50488 (**1**) is a weak agonist of the κ-opioid receptor.[5] Compound **1** represents an interesting starting point for a drug discovery programme, as it has potent antiproliferative activity against *T. brucei* (EC_50_=59 nm) with substantial selectivity over MRC-5 cells. It is also able to cross the blood–brain barrier (B/B ratio=8.2),[4] a key requirement to treat phase 2 HAT.

**Table Table 1:** Structure and EC_50_ values for (+)-(1*R*,2*R*)-U50488 and related compounds

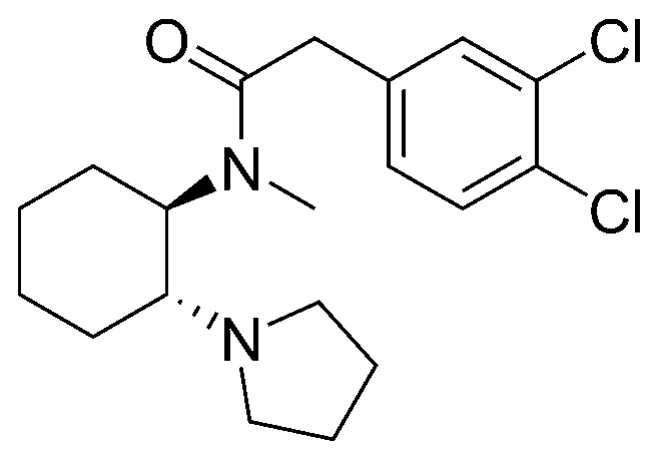			
Compd	Stereochemistry	EC_50_ [μm]	
		*T. brucei*	MRC-5
**1**	*R*,*R* (+)	0.028[a]	44
**2**	*S*,*S* (−)	9.9	48
**3**	*R*,*R*/*S*,*S* (±)	0.052	40

[a] Data acquired with our 384-well format assay (see Experimental Section), which is similar to data acquired with the original 96-well format assay.[4]

Herein we describe our studies to identify the key pharmacophoric elements of **1**, including the optimisation of *T. brucei* antiproliferative activity and selectivity over the mammalian MRC-5 cell line. Improvement in metabolic stability would also be beneficial. Compound **1** was identified as a hit from the initial screen.[4] No other structurally related compounds were present in the LOPAC. The alternative *trans* stereoisomer of U50488 was purchased and tested to determine if antiproliferative activity is stereospecific. It was found that the (+)-*R*,*R* stereoisomer **1** is ∼350-fold more potent than the (−)-*S*,*S* stereoisomer **2**, but roughly equipotent with the (±)-*R*,*R*/*S*,*S* racemate **3** (Table 1), suggesting that the less active stereoisomer does not antagonise the effects of the active stereoisomer. In light of this result, and to simplify our chemistry programme, we chose to synthesise racemic mixtures for our initial explorations of SAR. Enantiospecific syntheses of these compounds were reported previously and could be adapted for the synthesis of any hit compound, should it be developed further.[6, 7]

## Results and Discussion

### Chemistry

**Figure 1:**
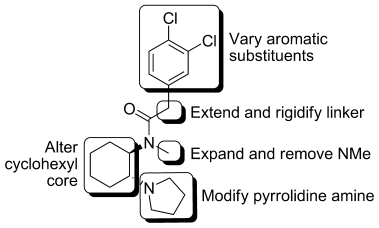
Planned alterations to U50488.

**Scheme 1:**
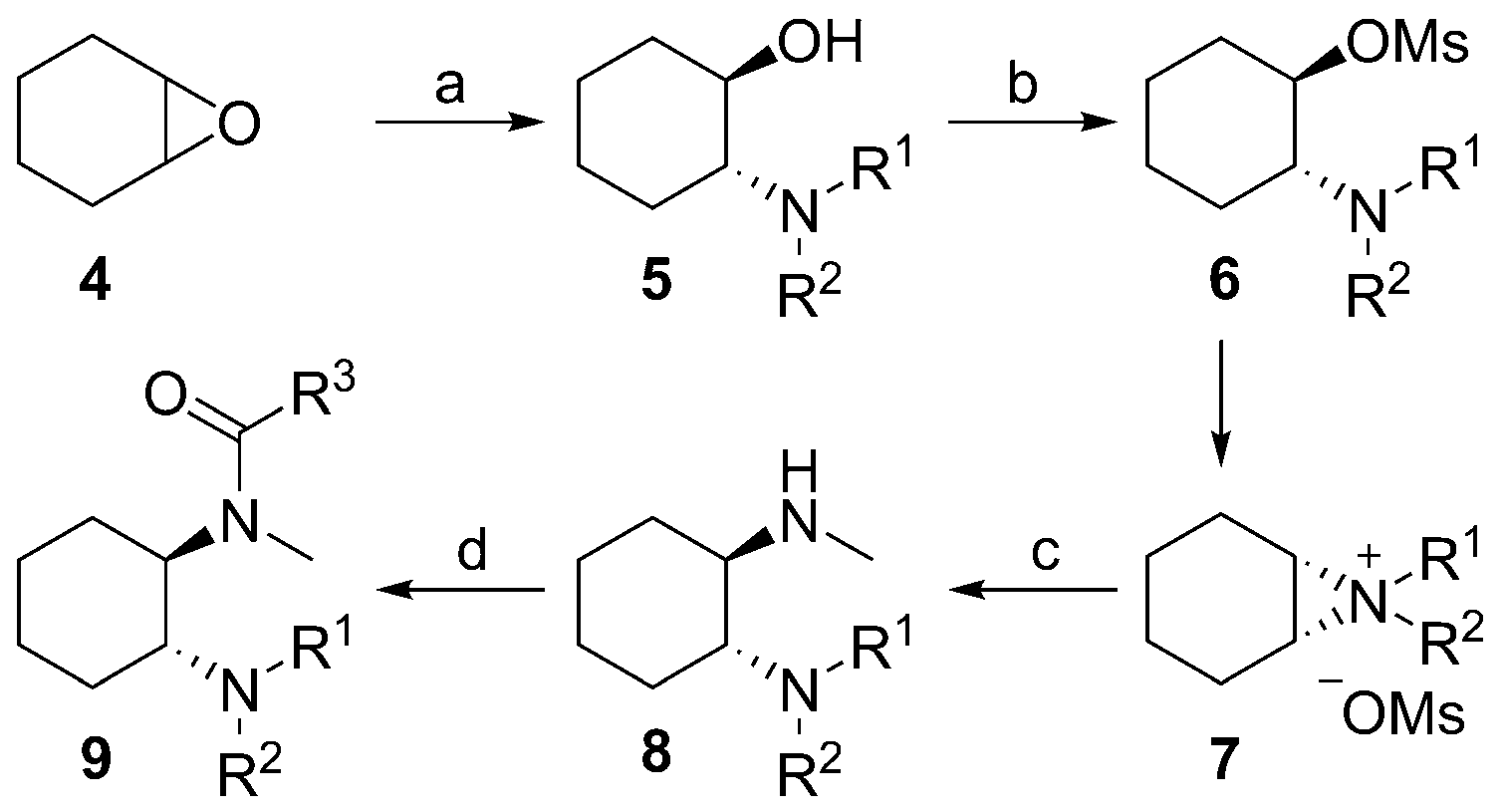
*Reagents and conditions:* a) NR^1^R^2^, EtOH/*i*PrOH, 80 °C, 18 h; b) MsCl/Et_3_N, 0 °C, 2 h; c) MeNH_2_, RT, 16 h; d) Bromotrispyrrolidinophosphonium hexafluorophosphate (PyBrop), Et_3_N, CH_2_Cl_2_, 30 min.

To probe the SAR of starting compound **1**, a number of modifications to the core were planned (Figure 1). Analogues of U50488 were prepared by using the chemistry outlined in Scheme 1. Cyclohexene oxide **4** was treated with an amine to effect epoxide ring opening. The resulting aminocyclohexanol **5** was treated with methanesulfonyl chloride to form mesylate **6**, which was displaced by the amine to form an aziridine ring **7**, which ring-opened upon reaction with methylamine to give diamine **8**.[6] The remaining free NH group was then allowed to react with a range of acids or acid chlorides to give the desired acetamides **9**.

### Biological assays

Initial investigations focussed on modification of the phenylacetamide moiety (Table 2). Removal of both chlorines to give the unsubstituted phenylacetamide **10** resulted in a dramatic loss of activity relative to **3**. Removal of either chlorine resulted in a six- to eightfold decrease in activity, as observed with compounds **11** and **12**, as did replacement with 3,4-difluorophenyl (compound **15**). Although this decrease could have been driven by the decrease in lipophilicity upon removal or replacement of the chlorines, the 2,4- and 2,6-dichloro substitution patterns in **14** and **13**, showing a respective six- and 210-fold loss in potency, indicate a structural element in the SAR. The *ortho*-methyl substituent in **20** was also poorly tolerated, perhaps suggesting a twisted conformation is not favourable. The 1-naphthyl group in **26** conferred some loss in activity relative to **3**, but the 2-naphthyl compound **27** is equipotent. The 2-naphthyl compound was, in fact, the only analogue in this subset to exhibit activity against the trypanosomes similar to that of the original 3,4-dichlorophenyl compound **3**, presumably with the second benzene ring occupying a similar space as the 3,4-dichloro substituents of the original molecule. On the whole, single substitutions at the 3- or 4-positions are roughly equipotent, as observed with the chlorophenyls (**11** and **12**), fluorophenyls (**16** and **17**), tolyls (**21** and **22**), and (trifluoromethyl)phenyls (**24** and **25**). The much larger biphenyl substituent in **28** was also tolerated; however, this compound showed the highest level of MRC-5 cell toxicity observed in this subset. Lastly, the aliphatic carbocycle **32** and the pyridyl **31** both suffered significantly lower activity than compound **3**.

**Table 2:** Phenyl substitution variation in compounds **10**–**32**

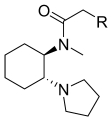					
Compd	R	EC_50_ [μm]		*CL*_i_ [mL min^−1^ g^−1^][b]	
		*T. b. brucei*[a]	MRC-5	Human	Mouse
**3**	3,4-dichlorophenyl	0.052	40	3.3	3.6
**10**	phenyl	11	>50	0.4	8.0
**11**	3-chlorophenyl	0.41	>50	0.6	14
**12**	4-chlorophenyl	0.33	>50	ND	ND
**13**	2,6-dichlorophenyl	11	>50	3.7	11
**14**	2,4-dichlorophenyl	0.33	>50	1.9	4.6
**15**	3,4-difluorophenyl	0.32	>50	1.2	1.8
**16**	3-fluorophenyl	1.3	>50	<0.5	9.9
**17**	4-fluorophenyl	1.0	>50	<0.5	11
**18**	3-methoxyphenyl	7.2	>50	1.2	9.8
**19**	4-methoxyphenyl	0.49	>50	1.9	5.9
**20**	2-tolyl	3.0	>50	0.6	9.1
**21**	3-tolyl	0.70	>50	2.8	20
**22**	4-tolyl	0.74	>50	ND	13
**23**	4-(isopropyl)phenyl	0.40	>50	ND	14
**24**	3-(trifluoromethyl)phenyl	0.12	>50	ND	3.8
**25**	4-(trifluoromethyl)phenyl	0.47	>50	ND	<0.5
**26**	1-naphthyl	0.34	>50	2.3	22
**27**	2-naphthyl	0.034	46	4.7	20
**28**	(1,1′-biphenyl)-4-yl	0.099	19	ND	2.7
**29**	3-bromophenyl	0.16	>50	ND	9.5
**30**	3-bromo-4-methoxyphenyl	0.090	>50	ND	6.5
**31**	3-pyridyl	9.8	>50	ND	ND
**32**	cyclohexyl	2.3	>50	ND	5.2

[a] Hill slopes in the range of 0.3–2.2.

[b] Microsomal intrinsic clearance; ND: not determined.

Encouragingly, the potency of these compounds against the human MRC-5 cell line remained low in general, with the majority exhibiting EC_50_>50 μm. Intrinsic clearance rates in mouse (the standard efficacy model species for HAT) are generally high, with nearly all compounds having higher metabolic instability than **3**, at 3.6 mL min^−1^ g^−1^. However, with human liver microsomes, the clearance rates are encouragingly much lower; the fluorophenyl compounds **16** and **17** are quite stable, showing rates of <0.5 mL min^−1^ g^−1^.

A small series of amines were synthesised to explore the SAR around the pyrrolidine in **3** (Table 3). Enlarging the ring size to 6 and 7 atoms (**33** and **34**) did not change the potency significantly. A more significant loss in potency was observed with the introduction of a second heteroatom to the ring in compounds **35** and **36**, as with the presence of *gem*-difluoro substituents on the pyrrolidine ring (compound **37**). In these compounds, the decreased basicity of the nitrogen atom could be the cause of this trend. Activity against MRC-5 cells remained low for these compounds (EC_50_>50 μm), except for the *N*-methylpiperazine **36**, with an EC_50_ value of 27 μm. Human intrinsic clearance measurements indicate that the larger alkylamine rings and the morpholine exhibit much lower metabolic stability than **3**, whereas the *N*-methylpiperazine shows a similar rate.

**Table 3:** Amine variation in compounds **33**–**37**

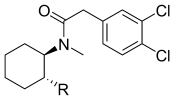					
Compd	R	EC_50_ [μm]		*CL*_i_ [mL min^−1^ g^−1^][b]	
		*T. b. brucei*[a]	MRC-5	Human	Mouse
**3**	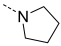	0.052	40	3.3	3.6
**33**	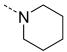	0.076	>50	11	ND
**34**	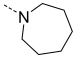	0.18	>50	15	--
**35**	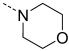	0.73	>50	29	ND
**36**	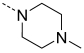	3.3	27	3.4	ND
**37**	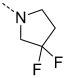	3.2	>50	ND	ND

[a] Hill slopes in the range of 1.2–3.9.

[b] Microsomal intrinsic clearance; ND: not determined.

Our next investigation focussed on the amide *N*-alkyl substituent (Table 4). This work demonstrated that the original *N*-methyl group is optimal, as its removal (compound **38**) led to a 10-fold loss in potency; its homologation (in **39** and **40**) resulted in a significant decrease in activity (80–400-fold). Both **38** and **41** showed an improvement in microsomal stability, suggesting that demethylation may be an issue in this regard.

**Table 4:** Modifications to the amide *N*-alkyl substituent in compounds **38**–**41**

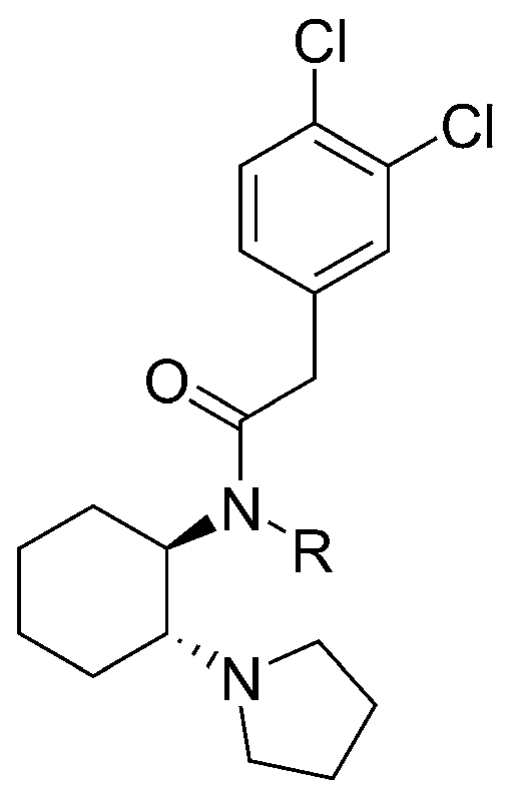					
Compd	R	EC_50_ [μm]		*CL*_i_ [mL min^−1^ g^−1^][b]	
		*T. b. brucei*[a]	MRC-5	Human	Mouse
**3**	methyl	0.052	40	3.3	3.6
**38**	H	0.27	28	2.6	1.8
**39**	ethyl	8.6	42	ND	ND
**40**	*tert*-butyl	11	50	ND	ND
**41**	benzyl	2.4	8.0	ND	1.1

[a] Hill slopes in the range of 1.3–8.9.

[b] Microsomal intrinsic clearance; ND: not determined.

We decided to investigate changes to the cyclohexyl ring to determine whether an increase in potency is possible here. In addition, one possible mechanism of metabolism is through hydroxylation of this ring. The corresponding tetrahydrofuranyl **42** and cyclopentyl **43** derivatives (Table 5) were synthesised by using the same chemistry as described in Scheme 1, starting with cyclopentyl and tetrahydrofuran oxide instead of cyclohexyl oxide. These modifications resulted in a significant loss in activity. Similarly, removal and replacement with an ethylene linker (to give **45**) resulted in a >60-fold decrease in potency, whilst replacement of the cyclohexyl ring with a planar phenyl ring in **44** also resulted in a >66-fold loss of potency relative to **38**. Interestingly, the introduction of a non-fused phenyl ring adjacent to the amide resulted in a stereochemically sensitive increase in activity, with the *R* isomer **46** demonstrating fivefold more potency that the *S* isomer **47**. Another strategy was to fuse the cyclohexyl ring to the amide nitrogen to form a piperidine ring and to extend the pyrrolidine with a methylene linker to produce **48**; this compound also showed poor activity.

**Table 5:** Core ring variation in compounds **42**–**48**

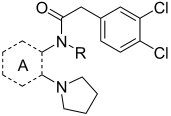						
Compd	Ring A	R	EC_50_ [μm]		*CL*_i_ [mL min^−1^ g^−1^][b]	
			*T. b. brucei*[a]	MRC-5	Human	Mouse
**3**	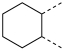	methyl	0.052	40	3.3	3.6
**42**	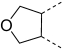	methyl	16.6	50	ND	ND
**43**	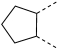	methyl	50	50	ND	ND
**44**	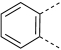	H	17.7	50	ND	ND
**45**	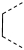	H	16.3	50	0.6	ND
**46**	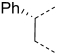	methyl	0.88	9.8	2.2	6.8
**47**	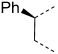	methyl	5.4	8.3	1.4	7.4
**48**	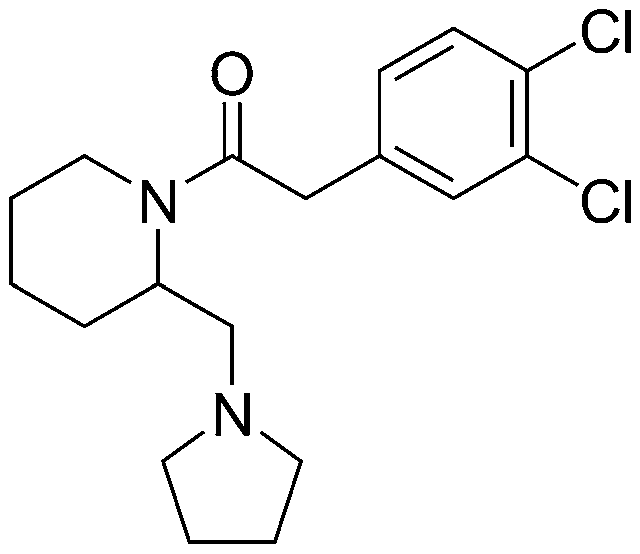	–	9.9	50	ND	ND

[a] Hill slopes in the range of 1.1–8.8.

[b] Microsomal intrinsic clearance; ND: not determined.

All changes to the cyclohexyl moiety led to a significant decrease in activity, suggesting that the stereochemistry and resulting conformation is very important for activity. This is consistent with the results obtained with (−)-(1*S*,2*S*)-U50488 (**2**), which shows a significant decrease in activity relative to the (+)-(1*R*,2*R*) enantiomer **1**.

To investigate the role of the linker between the amide carbonyl group and the phenyl ring, the linker was extended and also rigidified (Table 6). Removal of the methylene unit in the 3- and 4-biphenyls (**49** and **50**) demonstrated a significant loss in potency (compare with **28**). Addition of an oxygen atom (in **51**) was reasonably tolerated (sevenfold loss in activity). Chain extension to either two or three methylene units showed an increasing loss of potency (**52**: 11-fold; **53**: 27-fold). Rigidification through the introduction of cyclopropyl (in **54**) and cyclopentyl (in **55**) in place of the methylene linker showed a greater than fivefold improvement in potency over compound **10**, with the three-membered ring being better tolerated than the five. *gem*-Dimethyl **56** gave a sixfold increase in activity relative to compound **11**. Inclusion of the best elements from the SAR studies into one compound gave the 3,4-dichlorophenyl cyclopropyl analogue **57**, which demonstrated single-digit nanomolar inhibition of parasite growth in culture, with low levels of toxicity towards MRC-5 cells. Both this compound and the dimethyl analogue **56** showed similar or decreased clearance rates in human liver microsomes relative to parent compound **3**. Unfortunately, these compounds had higher mouse liver microsome clearance rates, which may make efficacy testing in a mouse model of infection problematic.

**Table 6:** Linker variation in compounds **49**–**57**

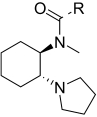					
Compd	R	EC_50_ [μm]		*CL*_i_ [mL min^−1^ g^−1^][b]	
		*T. b. brucei*[a]	MRC-5	Human	Mouse
**3**	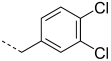	0.052	40	3.3	3.6
**49**	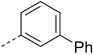	1.8	50	ND	12
**50**	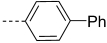	11	50	ND	ND
**51**	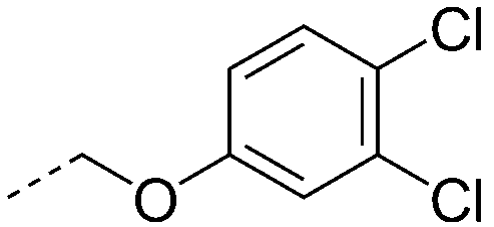	0.35	33	3.3	5.4
**52**	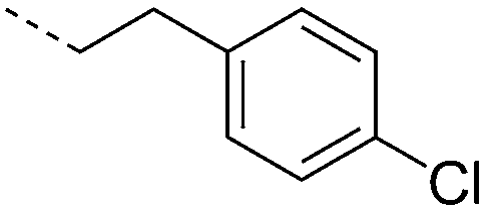	0.56	50	1.3	3
**53**	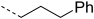	1.4	50	ND	5.3
**54**	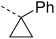	0.34	50	ND	25
**55**	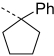	0.58	50	2.8	14
**56**	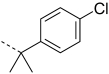	0.053	50	1.8	9.1
**57**	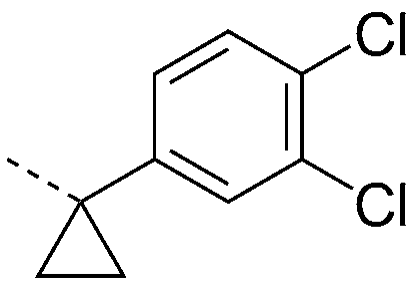	0.007	46	2.9	17

[a] Hill slopes in the range of 1.3–12.0.

[b] Microsomal intrinsic clearance; ND: not determined.

### Modelling

We were interested to investigate if there is a direct correlation between the activity of these molecules and their three-dimensional shape and physicochemical properties. Such a correlation could indicate a specific molecular target. Based on the similarity principle, which describes that similar molecules exhibit similar biological effects,[8] we first attempted to deduce the bioactive conformation of hit compound **1** by overlaying its multiple 3D conformations with multiple conformations of the most potent analogue **57**. Using ROCS software,[9] the conformation of **1** derived from the best-fit overlay of these two molecules was chosen as the bioactive conformation of the hit, and was subsequently used as the template conformation for further overlay experiments with other structural analogues within the compound series. Multiple conformations generated for the remaining 48 compounds were then overlaid with the proposed bioactive conformation of **1**, and ranked according to similarity in molecular shape and functional group complementarity relative to **1**.

**Figure 2:**
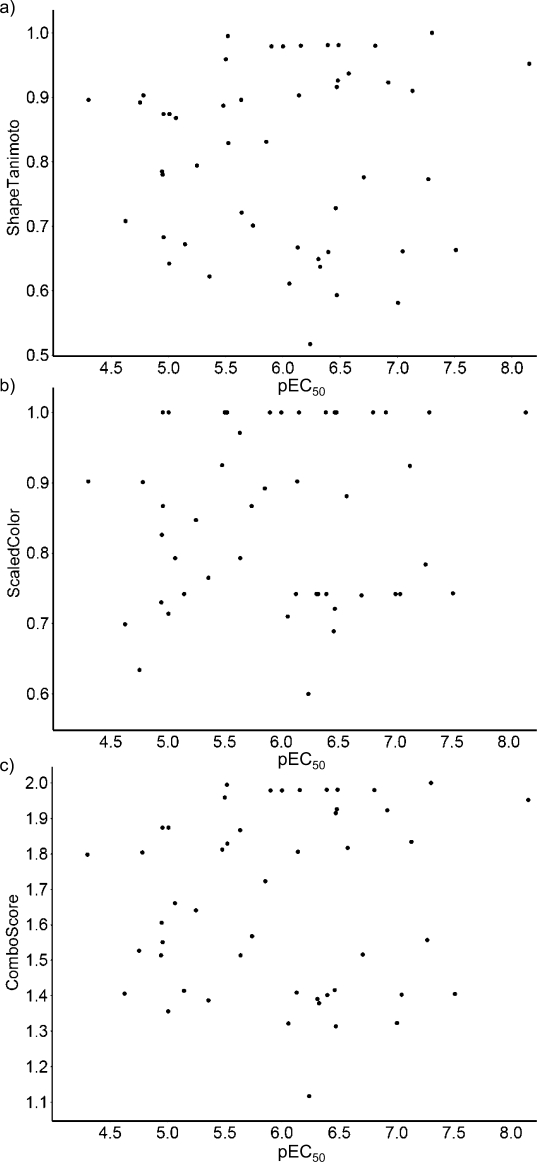
Plots of: a) molecular shape similarity (ShapeTanimoto score) and b) functional group complementarity (ScaledColor score) to hit compound **1** against potency values in *T. b. brucei* cells represented as pEC_50_. c) The ComboScore value plot represents the sum of the ShapeTanimoto and ScaledColor scores.[9]

Figure 2 a shows the plot of the potency values observed in *T. b. brucei* cells (pEC_50_) against the ShapeTanimoto score,[9] which describes the molecular shape resemblance of the structural analogues compared with hit compound **1**. Similarly, Figure 2 b shows the plot of pEC_50_ against the ScaledColor score,[9] which represents functional group complementarity to hit compound **1**. The higher the value of these scores, the more similar the compound is to hit compound **1**. It was apparent from these graphs that there is no correlation between either of these properties and the observed potency. This could suggest that the mechanism of inhibition of parasitic growth observed for these compounds might involve polypharmacological effects, which could also explain the very “tight” SAR. Alternatively, the active conformation of compound **1** might be significantly different from the predicted conformation.

## Conclusions

(+)-(1*R*,2*R*)-U50488 (**1**) is an exciting starting point for a medicinal chemistry programme for HAT, as it has good in vitro potency against *T. brucei*, acceptable in vitro and in vivo drug metabolism and pharmacokinetics (DMPK) properties, and is blood–brain barrier penetrant (B/B ratio=8.2).[4] Our previous studies[4] indicated that the compound has good oral exposure, a half-life in mice of 2.6 h and a large unbound fraction (0.69). It is possible to obtain concentrations above EC_99_ in mice for 4 h following oral dosing of 150 mg kg^−1^. Unfortunately it was not curative in mice under a dosing regimen that maintained levels above EC_99_ for 32 h. The reasons for this are not entirely clear, but are probably due to the compound only being cytocidal at high concentrations. Furthermore, **1** displays a relatively low Hill slope (*s*=1.3), therefore requiring >10 times the EC_50_ to obtain EC_99_. This contrasts with eflornithine, a cytostatic HAT drug (*s*=4.0) which requires only three times its EC_50_ to obtain EC_99_.[4] Therefore, (+)-(1*R*,2*R*)-U50488 (**1**) requires further optimisation to achieve efficacy.

Herein we report studies to optimise potency and to systematically generate structure–activity relationships. Further improvement in metabolic stability would also be beneficial. Our investigations across all moieties of the molecule showed that it displays very tight SAR, but there are some possibilities for increasing the potency. Alternative aromatic groups to the 3,4-dichlorophenyl in the original hit are reasonably well tolerated, although the activity was not significantly improved. The conformation of the central cyclohexyl ring appears to be vital for activity, as modification of the core resulted in significant loss of potency. Other changes to the pyrrolidine ring and amide N substituent resulted in loss of activity. The greatest gain in potency was achieved by rigidifying the methylene linker of the amide moiety, which gave compound **57** (EC_50_=0.007 μm), with a sevenfold increase in potency relative to the parent compound **3** (EC_50_=0.052 μm). Compound **57** has similar metabolic stability in human microsomes to that of the parent. However, there is a decrease in mouse microsomal stability, which could make efficacy testing problematic. This compound has a similar Hill slope to that of **3** (**3**: *s*=1.5; **57**: *s*=1.4), indicating that a large concentration of compound would be required to obtain the EC_99_ in vivo.

Molecular modelling experiments were employed to compare the molecular shape and functional group complementarity of structural analogues with hit compound **1**. Assessments of these calculations revealed little correlation between the observed potency and either of these properties. This could suggest that the compounds do not inhibit just a single molecular target, but show more complex polypharmacological behaviour. Alternatively, the inhibitors may bind in an unexpected conformation.

We have established that it is possible to increase the potency of the lead compound, although there are relatively limited options for modification of the parent structure. Further work to determine the molecular target(s) of the compound series may indicate further strategies to increase potency and allow scope for improving the DMPK properties of the molecules.

## Experimental Section

### Biology

Growth inhibition was determined using *T. brucei* (S427 single marker line). *T. brucei* culture (50 μL at a density of 1×10^4^ cells mL^−1^ in HMI9-T media)[4] was added, using a Wellmate (ThermoMatrix), to 384-well plates containing test compounds (250 nL at 2 nm to 50 μm final concentration), yielding a final DMSO concentration of 0.5 %. Columns 11 and 12 of the plate were used as full signal control wells (0 % inhibition) and columns 23 and 24 as low signal control (100 % inhibition). Plates were then incubated at 37 °C in a humidified incubator with an atmosphere of 5 % CO_2._ After 68 h, Resazurin (5 μL of 500 μm stock to give a final concentration of 45 μm) was added to each well, and the plates were incubated for a further 4 h before being measured for fluorescence (*λ*_ex_=528 nm, *λ*_em_=590 nm). The fluorescence signal for each well was background subtracted and expressed as a percentage of inhibition relative to the full signal control wells.

A counter-screen against normal diploid human fibroblasts (MRC-5 cell line) was carried out to exclude nonselective compounds. Cells (80 μL) were plated at a density of 1.25×10^4^ cells mL^−1^ into wells 1–22 of a 384-well plate and incubated overnight to allow them to adhere as monolayers. A working stock (2 μL of 2 nm to 50 μm final concentration) of each test compound was transferred to an intermediate 384-well plate using a Matrix Platemate Plus (Thermo) and pre-diluted with 38 μL minimum essential media (MEM). The pre-diluted stocks (9 μL) were then transferred onto the cell monolayers using a Beckman FX instrument, and the plates were incubated for 68 h as described above. After 68 h, Resazurin (10 μL of 500 μm stock to give a final concentration of 50 μm) was added to each well, plates were incubated and measured for fluorescence as described for the *T. brucei* assay.

EC_50_ curve fitting for both cell types employed a four-parameter logistic dose–response curve using IDBS XLfit 4.2 Model 205 [Eq. (1)]:


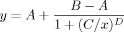
(1)

For which *A* is the minimum *y* value, B is the maximum *y* value, *C* is the EC_50_ and *D* is the Hill slope. All test compounds had floating maximum and minimum, and pre-fit was used for all four parameters. Pentamidine was used as a standard comparator drug for *T. brucei* in all experiments (pEC_50_=8.45±0.38, *n*=63), and doxorubicin as a standard comparator drug for MRC-5 (pEC_50_=7.15±0.55, *n*=47).

### Drug metabolism and pharmacokinetics (DMPK)

Each test compound (0.5 μm) was incubated with female CD1 mouse or pooled human liver microsomes (Tebu-Bio, UK; 0.5 mg mL^−1^ in 50 mm potassium phosphate buffer, pH 7.4), and the reaction was started with the addition of excess NADPH (8 mg mL^−1^ in 50 mm potassium phosphate buffer, pH 7.4). Immediately at time zero, then at 3, 6, 9, 15, and 30 min, aliquots (50 μL) of the incubation mixture were removed and mixed with MeCN (100 μL) to stop the reaction. Internal standard was added to all samples, the samples were centrifuged (1800 *g*, 4 °C, 10 min) to sediment precipitated protein, and the plates were sealed prior to UPLC–MS–MS analysis using a Quattro Premier XE instrument (Waters).

XLfit (IDBS, UK) was used to calculate the exponential decay and consequently the rate constant (*k*) from the ratio of peak area of test compound to internal standard at each time point. The rate of intrinsic clearance (*CL*_i_, [mL min^−1^ (g liver)^−1^]) of each compound was then calculated [Eq. 2]:


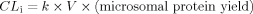
(2)

For which *V* [mL (mg protein)^−1^] is the incubation volume per mg protein added, and microsomal protein yield is taken as 52.5 mg protein per g liver. Verapamil was used as a positive control to confirm acceptable assay performance.

### Molecular modelling

Multiple 3D conformations of all compounds were generated using OMEGA2 (OpenEye Scientific Software Inc.), with a threshold RMS distance of 0.5 per conformer, and a maximum of 200 conformers for each molecule; 92 conformations were selected for hit compound **1**, and 32 conformations for analogue **57** in the initial experiment to determine the bioactive conformation of compound **1**. The number of conformations for all other analogues ranged from one single conformer (compound **55**) to the maximum number allowed of 200 (compounds **37** and **45**–**47**).

Structural overlay was performed using ROCS[9] (OpenEye Scientific Software Inc.) by applying the ImplicitMillsDean force field as the Color force field for functional group complementarity comparisons. Compounds were ranked by ComboScore, which represented a combination of the ShapeTanimoto and the ScaledColor scores.

### Chemistry

^1^H NMR spectra were recorded on a Bruker Avance DPX 500 instrument. Chemical shifts (*δ*) are expressed in ppm. Signal splitting patterns are described as singlet (s), broad singlet (bs), doublet (d), triplet (t), quartet (q), multiplet (m), or combination thereof. Low-resolution electrospray (ES) mass spectra were recorded on a Bruker MicroTof mass spectrometer, run in positive ion mode, using either MeOH, MeOH/H_2_O (95:5) or H_2_O/MeCN (1:1) and 0.1 % formic acid as the mobile phase. High-resolution electrospray MS measurements were performed on a Bruker MicroTof mass spectrometer. LC–MS analyses were performed with an Agilent HPLC 1100 instrument (Phenomenex Gemini Column 5 μm C_18_ 110A 50×3.0 mm, eluted with (0–3 min 20 % MeOH/H_2_O) and a diode array detector in series with a Bruker MicroTof mass spectrometer. All synthesised compounds were determined to be of >95 % purity by LC–MS. Thin-layer chromatography (TLC) was carried out on Merck silica gel 60 F_254_ plates using UV light and/or KMnO_4_ for visualisation. Column chromatography was performed using RediSep 4 or 12 g silica pre-packed columns. All reactions were carried out under dry and inert conditions unless otherwise stated.

Experimental details for key compounds and some intermediates are included herein; specifics for other compounds are included in the Supporting Information.

**General procedure for diamine formation 8:** Cyclohexene oxide (1 mL, 10 mmol) and amine (12 mmol) in EtOH (8 mL) were heated by microwave at 140 °C for 40 min, concentrated to dryness, and used without further purification. Methanesulfonyl chloride (MsCl; 1 mL, 12 mmol) was added dropwise to an ice-cold solution of the resulting aminocyclohexanol and Et_3_N (4.39 mL, 30 mmol) in anhydrous Et_2_O (10 mL). The reaction mixture was stirred for 10 min, methylamine (41 % aqueous solution, 5 mL) was added, and the reaction mixture was allowed to warm to room temperature with vigorous stirring for 18 h. The layers were separated, the aqueous layer further extracted with Et_2_O (150 mL), and the combined organics were dried over MgSO_4_ and concentrated in vacuo. The resulting gum was purified by column chromatography to give **8**.

***trans*****-(±)-2-(Pyrrolidin-1-yl)cyclohexanol (5):** Pyrrolidine (13.37 mL, 162 mmol) was added to a solution of cyclohexene oxide (10 mL, 99 mmol) in EtOH (100 mL). The reaction mixture was stirred at reflux for 18 h, then concentrated to dryness in vacuo to give the title compound in quantitative yields. The resulting material was used without further purification. ^1^H NMR (300 MHz, CDCl_3_): *δ*=4.00 (bs, 1 H), 3.28–3.37 (m, 1 H), 2.61–2.72 (m, 2 H), 2.50–2.60 (m, 2 H), 2.39–2.50 (m, 1 H), 2.04–2.14 (m, 1 H), 1.63–1.83 (m, 7 H), 1.10–1.33 ppm (m, 4 H).

***trans*****-(±)-*N*-Methyl-2-(pyrrolidin-1-yl)cyclohexanamine (8 a):** MsCl (9.14 mL, 117 mmol) was added dropwise to an ice-cold solution of *trans*-(±)-2-(pyrrolidin-1-yl)cyclohexanol (99 mmol) and Et_3_N (41.4 mL, 297 mmol) in anhydrous Et_2_O (150 mL). The reaction mixture was stirred for 30 min, methylamine (41 % aqueous solution, 48 mL) was added, and the reaction mixture was allowed to warm to room temperature with vigorous stirring for 18 h. The layers were separated, the aqueous layer further extracted with Et_2_O (150 mL), and the combined organics were dried over MgSO_4_ and concentrated in vacuo. The resulting gum was purified by column chromatography to give 13.5 g of the title compound (75 %). ^1^H NMR (500 MHz, CDCl_3_): *δ*=2.54–2.60 (m, 2 H), 2.49–2.54 (m, 2 H), 2.41–2.47 (m, 1 H), 2.38 (s, 3 H, CH_3_), 2.13–2.19 (m, 1 H), 2.05–2.11 (m, 1 H), 1.74–1.79 (m, 2 H), 1.64–1.73 (m, 5 H), 1.13–1.26 (m, 3 H), 0.93–1.02 ppm (m, 1 H); LCMS *m*/*z*: 183 [*M*+H]^+^, *t*_R_*=*0.6–0.8 min.

#### General procedure for amide formation from 8 with an acid or acid chloride 9

**Method A:** A flask was charged with **8** (1 mol equiv), acid (2 mol equiv), and HOBt (2 mol equiv) in DMF (anhydrous, 2 mL mmol^−1^). DIPEA (2 mol equiv) and EDCI (2 mol equiv) were added, and the reaction mixture was stirred at room temperature for 1.5–4 h. After concentration in vacuo, the resulting residue was partitioned between EtOAc and a saturated aqueous NaHCO_3_ solution, the organics were dried over MgSO_4_, concentrated, and purified by column chromatography. The resulting gum was taken up in EtOAc/Et_2_O (1:2), 2 m HCl in Et_2_O (1–2 mL) was added dropwise, and the HCl salt of **9** was collected by filtration.

**Method B:** Acid chloride (1.1 mol equiv) was added to an ice-cold solution of **8** (1 mol equiv) in CH_2_Cl_2_ (anhydrous, 2 mL mmol^−1^). The reaction mixture was allowed to warm to room temperature and stirred for a total of 2 h, then diluted with further CH_2_Cl_2_, washed with a saturated aqueous NaHCO_3_ solution, and the organics were dried over MgSO_4_ and concentrated. An HCl salt of **3** was obtained as in Method A.

**Method C:** Compound **8** (1 mol equiv), acid (1 mol equiv), Et_3_N (0.4 mL mmol^−1^), and PyBrop (1.2 mol equiv) in CH_2_Cl_2_ (2 mL mmol^−1^) were stirred under argon for 40 min at 4 °C. The reaction was diluted with CH_2_Cl_2_ and washed with H_2_O (3×10 mL). Purification by column chromatography eluting with CH_2_Cl_2_/MeOH 95:5 afforded the desired products.

**2-(3,4-Dichlorophenyl)-*N*-methyl-*N*-(*trans*-(±))-[2-(pyrrolidin-1-yl)cyclohexyl]acetamide⋅HCl (3):** Prepared according to Method A from *trans*-(±)-*N*-methyl-2-(pyrrolidin-1-yl)cyclohexanamine (100 mg, 0.55 mmol), 3,4-dichlorophenylacetic acid (226 mg, 1.1 mmol), HOBt (149 mg, 1.1 mmol), DIPEA (192 μL, 1.1 mmol), and EDCI (211 mg, 1.1 mmol) in DMF (anhydrous, 5 mL) in 56 % yield. ^1^H NMR (500 MHz, [D_6_]DMSO): *δ*=9.58 (bs, HCl), 7.55 (d, 1 H, ArH, *J*=8.3 Hz), 7.52 (d, 1 H, *J*=1.8 Hz, ArH), 7.24 (dd, 1 H, ArH, *J*=8.3 and 1.9 Hz), 4.52 (bs, 1 H), 3.93 (d, 1 H, *J*=16.2 Hz), 3.72 (d, 1 H, *J*=16.4 Hz), 3.53–3.62 (m, 1 H), 3.41–3.48 (m, 1 H), 3.10–3.28 (m, 3 H), 2.94 (s, 3 H, CH_3_), 2.02–2.10 (m, 1 H), 1.80–1.96 (m, 4 H), 1.73–1.80 (m, 1 H), 1.65–1.72 (m, 1 H), 1.46–1.63 (m, 3 H), 1.21–1.36 ppm (m, 2 H); ^13^C NMR (125 MHz, [D_6_]DMSO): *δ*=171.4, 137.6, 131.9, 130.5, 130.2, 129.9, 128.7, 60.1, 51.4, 47.7, 39.2, 28.6, 24.2, 23.9, 23.4, 23.0 ppm; LC–MS *m*/*z*: 369 [*M*+H]^+^, *t*_R_*=*3.9 min; HRMS (ESI) calcd for C_19_H_27_N_2_OCl_2_ 369.1500 [*M*+H]^+^, found 369.1508.

***N*****-Methyl-2-(naphthalen-2-yl)-*N*-(*trans*-(±))-[2-(pyrrolidin-1-yl)cyclohexyl]acetamide⋅HCl (27):** Prepared according to Method A from *trans*-(±)-*N*-methyl-2-(pyrrolidin-1-yl)cyclohexanamine (100 mg, 0.55 mmol), 2-(naphthalen-2-yl)acetic acid (205 mg, 1.1 mmol), HOBt (149 mg, 1.1 mmol), DIPEA (192 μL, 1.1 mmol), and EDCI (211 mg, 1.1 mmol) in DMF (anhydrous, 5 mL) in 35 % yield. ^1^H NMR (500 MHz, CDCl_3_): *δ*=11.39 (bs, 1 H, HCl), 7.72–7.81 (m, 4 H, ArH), 7.40–7.48 (m, 3 H, ArH), 4.66–4.87 (m, 1 H), 4.25 (d, 1 H, *J*=15.9 Hz), 4.05 (d, 1 H, *J*=15.9 Hz), 3.88–3.98 (m, 1 H), 3.60–3.68 (m, 1 H), 3.25–3.39 (m, 1 H), 3.10 (s, 3 H, CH_3_), 2.96–3.07 (m, 2 H), 2.08–2.30 (m, 3 H), 1.90–1.98 (m, 1 H), 1.74–1.88 (m, 4 H), 1.54–1.64 (m, 1 H), 1.44–1.54 (m, 1 H), 1.33–1.43 (m, 1 H), 1.24–1.33 ppm (m, 1 H); ^13^C NMR (125 MHz, [D_6_]DMSO): *δ*=172.1, 133.8, 132.9, 131.7, 128.4, 127.6, 127.4, 127.3, 127.2, 125.9, 125.4, 60.0, 51.2, 47.8, 40.7, 39.7, 28.7, 24.1, 24.0, 23.8, 23.4, 23.3 ppm; LC–MS *m*/*z*: 351 [*M*+H]^+^, *t*_R_*=*3.8 min; HRMS (ESI) calcd for C_23_H_31_N_2_O 351.2436 [*M*+H]^+^, found 351.2434.

**2-(3,4-Dichlorophenyl)-*N*-methyl-*N*-(*trans*-(±))-(2-morpholinocyclohexyl)acetamide⋅HCl (35):** Prepared according to Method A from *trans*-(±)-*N*-methyl-2-morpholinocyclohexanamine (100 mg, 0.5 mmol), 3,4-dichlorophenylacetic acid (205 mg, 1.0 mmol), HOBt (135 mg, 1.0 mmol), DIPEA (174 μL, 1.0 mmol), and EDCI (192 mg, 1.0 mmol) in DMF (anhydrous, 5 mL) in 75 % yield. ^1^H NMR (500 MHz, CDCl_3_): *δ*=11.58 (bs, 1 H, HCl), 7.40 (d, 1 H, ArH, *J*=2.1 Hz), 7.37 (d, 1 H, ArH, *J*=8.3 Hz), 7.20 (dd, 1 H, ArH, *J*=8.3 and 2.1 Hz), 5.02–5.09 (m, 1 H), 4.80–4.88 (m, 1 H), 4.28–4.34 (m, 1 H), 4.25 (d, 1 H, *J*=16.4 Hz), 3.94–3.99 (m, 1 H), 3.83–3.91 (m, 2 H), 3.65 (d, 1 H, *J*=16.4 Hz), 3.21–3.30 (m, 1 H), 3.12–3.19 (m, 2 H), 3.12 (s, 3 H, CH_3_), 2.86–2.94 (m, 1 H), 2.21–2.26 (m, 1 H), 1.97–2.02 (m, 1 H), 1.82–1.90 (m, 2 H), 1.59–1.69 (m, 1 H), 1.48–1.56 (m, 2 H), 1.25–1.42 ppm (m, 2 H); ^13^C NMR (125 MHz, [D_6_]DMSO): *δ*=171.9, 137.4, 131.8, 130.3, 130.3, 130.0, 128.8, 63.4, 62.6, 50.5, 48.8, 47.2, 30.3, 29.2, 23.9, 23.6 ppm; LC–MS *m*/*z*: 385 [*M*+H]^+^, *t*_R_*=*4.2 min; HRMS (ESI) calcd for C_19_H_27_N_2_O_2_Cl_2_ 385.1450 [*M*+H]^+^, found 385.1448.

**2-(3,4-Dichlorophenyl)-*N*-(*trans*-(±))-[2-(pyrrolidin-1-yl)cyclohexyl]acetamide (38):** Prepared according to Method A from *trans*-(±)-2-(pyrrolidin-1-yl)cyclohexanamine (100 mg, 0.59 mmol), 3,4-dichlorophenylacetic acid (242 mg, 1.18 mmol), HOBt (159 mg, 1.18 mmol), DIPEA (205 μL, 1.18 mmol), and EDCI (226 mg, 1.18 mmol) in DMF (anhydrous, 5 mL) in 14 % yield. ^1^H NMR (500 MHz, [D_6_]DMSO): *δ*=7.81 (bd, 1 H, NH, *J*=8.2 Hz), 7.54 (d, 1 H, ArH, *J*=8.2 Hz), 7.51 (d, 1 H, ArH, *J*=2.1 Hz), 7.26 (dd, 1 H, ArH, *J*=8.2 and 2.1 Hz), 3.61–3.68 (m, 1 H), 3.46 (d, 1 H, *J*=14.0 Hz), 3.37 (d, 1 H, *J*=14.0 Hz), 2.50–2.55 (m, 2 H), 2.35–2.46 (m, 3 H), 1.79–1.85 (m, 1 H), 1.72–1.78 (m, 1 H), 1.63–1.70 (m, 1 H), 1.46–1.63 (m, 5 H), 1.13–1.30 ppm (m, 4 H); ^13^C NMR (125 MHz, [D_6_]DMSO): *δ*=168.3, 138.1, 130.8, 130.6, 130.1, 129.3, 128.8, 61.4, 49.7, 47.5, 41.5, 31.4, 23.7, 23.7, 23.3 ppm; LC–MS *m*/*z*: 355 [*M*+H]^+^, *t*_R_*=*3.8 min; HRMS (ESI) calcd for C_18_H_25_N_2_OCl_2_ 355.1344 [*M*+H]^+^, found 355.1348.

**2-(3,4-Dichlorophenyl)-*N*-[2-(pyrrolidin-1-yl)phenyl]acetamide (44):** Prepared according to Method A from 2-(pyrrolidin-1-yl)aniline **8 b** (200 mg, 1.23 mmol), 3,4-dichlorophenylacetic acid (380 mg, 1.85 mmol), HOBt (250 mg, 1.85 mmol), DIPEA (322 μL, 1.85 mmol), and EDCI (355 mg, 1.85 mmol) in DMF (anhydrous, 5 mL) in 8 % yield. ^1^H NMR (500 MHz, CDCl_3_): *δ*=8.27–8.33 (m, 2 H, ArH, NH), 7.44–7.50 (m, 2 H, ArH), 7.18–7.23 (m, 1 H, ArH), 7.06–7.11 (m, 2 H, ArH), 7.00–7.05 (m, 1 H, ArH), 3.73 (s, 2 H), 2.71–2.79 (m, 4 H), 1.72–1.79 ppm (m, 4 H); ^13^C NMR (125 MHz, [D_6_]DMSO): *δ*=168.2, 143.8, 137.1, 131.3, 130.7, 130.4, 129.7, 129.3, 127.0, 126.6, 125.8, 119.3, 116.2, 50.1, 41.7, 24.5 ppm; LC–MS *m*/*z*: 349 [*M*+H]^+^, *t*_R_*=*5.1 min; HRMS (ESI) calcd for C_18_H_19_N_2_OCl_2_ 349.0874 [*M*+H]^+^, found 349.0861.

**2-(Pyrrolidin-1-yl)aniline (8 b):** 1-Chloro-2-nitrobenzene (1.25 g, 7.9 mmol) was stirred in neat pyrrolidine (20 mL) at room temperature for 6 h, then concentrated in vacuo. The residue was partitioned between EtOAc and saturated aqueous NaHCO_3_ solution, the organics were dried over MgSO_4_, concentrated, and the resulting bright-yellow oil was used without further purification. The formed 1-(2-nitrophenyl)pyrrolidine was subjected to standard hydrogenation conditions in EtOH (50 mL) with 10 % Pd/C (800 mg). The reaction mixture was stirred at room temperature under hydrogen balloon for 72 h, filtered through Celite, and the filtrate was concentrated. The resulting oil was taken up in CH_2_Cl_2_, dried over MgSO_4_, and concentrated to give an oil in near quantitative yield. ^1^H NMR (500 MHz, CDCl_3_): *δ*=6.98–7.01 (m, 1 H, ArH), 6.87–6.91 (m, ArH, 1 H), 6.72–6.76 (m, 2 H, ArH), 3.89 (bs, 2 H, NH_2_), 3.02–3.09 (m, 4 H), 1.89–1.96 ppm (m, 4 H); LC–MS *m*/*z*: 163 [*M*+H]^+^, *t*_R_*=*0.7 min.

**(*R*)-2-(3,4-Dichlorophenyl)-*N*-methyl-*N*-[1-phenyl-2-(pyrrolidin-1-yl)ethyl]acetamide⋅HCl (46):** 2-(3,4-Dichlorophenyl)acetic acid (402 mg, 1.96 mmol) was stirred in excess thionyl chloride at room temperature for 10 min, and then concentrated in vacuo to form the corresponding acid chloride. (*R*)-*N*-Methyl-1-phenyl-2-(pyrrolidin-1-yl)ethanamine (200 mg, 0.98 mmol) in CH_2_Cl_2_ (3 mL) was added slowly to a stirring solution of the acid chloride in CH_2_Cl_2_ (30 mL) at 0 °C. DIPEA (325 μL, 1.96 mmol) was added, and the reaction mixture was heated at 40 °C for 18 h. After concentrating in vacuo, the resulting residue was partitioned between CH_2_Cl_2_ and 2 n NaOH, the organics were dried over MgSO_4_, concentrated, and purified by column chromatography. The resulting gum was taken up in Et_2_O, 2 m HCl in Et_2_O was added dropwise, and the HCl salt was collected by filtration in 20 % yield. ^1^H NMR (500 MHz, [D_6_]DMSO): *δ*=9.99 (bs, 1 H, HCl), 7.57 (d, 1 H, ArH, *J*=8.2 Hz), 7.56 (d, 1 H, ArH, *J*=1.9 Hz), 7.41 (t, 2 H, ArH, *J*=7.4 Hz), 7.35 (t, 1 H, ArH, *J*=7.4 Hz), 7.29 (dd, 1 H, ArH, *J*=8.2 and 1.9 Hz), 7.26 (d, 2 H, ArH, *J*=7.4 Hz), 6.13 (dd, 1 H, *J*=12.1 and 2.6 Hz), 4.05–4.12 (m, 1 H), 3.94 (d, 1 H, *J*=16.3 Hz), 8.34 (d, 1 H, 16.3 Hz), 3.61–3.70 (m, 2 H), 3.50–3.57 (m, 1 H), 3.10–3.24 (m, 2 H), 2.78 (s, 3 H, CH_3_), 1.88–2.06 ppm (m, 4 H); ^13^C NMR (125 MHz, [D_6_]DMSO): *δ*=171.9, 137.5, 136.5, 132.0, 130.6, 130.2, 129.9, 128.8, 128.7, 128.0, 127.2, 55.1, 52.5, 51.1, 38.9, 29.8, 23.0, 22.6 ppm; LC–MS *m*/*z*: 391 [*M*+H]^+^, *t*_R_*=*4.1 min; HRMS (ESI) calcd for C_21_H_25_N_2_OCl_2_ 391.1344 [*M*+H]^+^, found 391.1329.

**1-Phenyl-*N*-[*trans*-(±)-2-(pyrrolidin-1-yl)cyclohexyl]cyclopropanecarboxamide (54):** Prepared according to Method C from *trans*-(±)-*N*-methyl-2-(pyrrolidin-1-yl)cyclohexanamine (91 mg, 0.5 mmol), 1-phenylcyclopropane carboxylic acid (81 mg, 0.5 mmol), Et_3_N (0.2 mL), and PyBrop (312 mg, 0.6 mmol) in CH_2_Cl_2_ (anhydrous, 1 mL). The crude residue was purified by column chromatography eluting with CH_2_Cl_2_/MeOH/NH_3_ 95:5:0.1 to afford **54** (86 mg, 53 %). ^1^H NMR (500 MHz, [D_6_]DMSO): *δ*=8.62 (bs, 1 H), 7.15 (t, 2 H, *J*=7.6 Hz, PhH), 7.04 (t, 1 H, *J*=7.3 Hz, PhH), 6.98 (d, 2 H, *J*=7.3 Hz, PhH), 4.35 (t, 1 H, *J*=10.5 Hz), 3.42 (t, 1 H, *J*=11.5), 3.08–3.05 (m, 2 H), 2.98–2.89 (m, 2 H), 2.45 (s, 3 H, CH_3_), 1.91 (d, 1 H), 1.67–1.64 (m, 3 H), 1.58–1.55 (m, 2 H), 1.49 (d, 1 H, *J*=12.3 Hz), 1.35–1.27 (m, 5 H), 1.24 (s, 2 H), 1.16–1.09 (m, 1 H), 1.04–0.97 ppm (m, 1 H); ^13^C NMR (125 MHz, [D_6_]DMSO): *δ*=172.8, 139.9, 128.8, 126.3, 125.4, 62.0, 59.7, 51.0, 49.3, 30.3, 28.4, 24.7, 23.9, 23.5, 23.1, 22.9, 16.1, 13.1 ppm; LC–MS: *m*/*z* 327 [*M*+H]^+^, *t*_R_=5.1–5.2 min; HRMS (ESI) calcd for C_21_H_31_N_2_O 327.2431 [*M*+H]^+^, found 327.2425.

***trans*****-(±)-1-Phenyl-*N*-[*trans*-(±)-2-(pyrrolidin-1-yl)cyclohexyl]cyclopentanecarboxamide (55):** Prepared according to Method C from *trans*-(±)-*N*-methyl-2-(pyrrolidin-1-yl)cyclohexanamine (91 mg, 0.5 mmol), 1-phenylcyclopentanecarboxylic acid (95 mg, 0.5 mmol), Et_3_N (0.2 mL), and PyBrop (312 mg, 0.6 mmol) in CH_2_Cl_2_ (anhydrous, 1 mL). The crude residue was purified by column chromatography eluting with CH_2_Cl_2_/MeOH/NH_3_ 95:5:0.1 to afford **55** (74 mg, 42 %). ^1^H NMR (500 MHz, [D_6_]DMSO): *δ*=8.79 (bs, 1 H), 7.28 (t, 2 H, *J*=7.7 Hz, PhH), 7.17 (t, 1 H, *J*=7.4 Hz, PhH), 7.12 (d, 2 H, *J*=7.45 Hz, PhH), 4.54 (bs, 1 H), 3.50 (bs, 1 H), 3.09–2.90 (m, 2 H), 2.39–2.33 (m, 1 H), 2.28 (s, 3 H, CH_3_), 2.25–2.21 (m, 1 H), 2.18–2.13 (m, 1 H), 2.04–2.02 (m, 1 H), 1.84–1.78 (m, 3 H), 1.70–1.57 (m, 6 H), 1.54–1.45 (m, 5 H), 1.34–1.32 (m, 2 H), 1.24–1.16 (m, 1 H), 1.11–1.05 ppm (m, 1 H); ^13^C NMR (125 MHz, [D_6_]DMSO): *δ*=172.1, 153.9, 133.7, 129.9, 129.3, 112.3, 110.1, 60.1, 56.1, 51.4, 48.5, 38.1, 28.6, 27.4, 24.3, 24.1, 23.9, 23.6, 23.5, 23.4, 23.3, 23.0 ppm; LC–MS: *m*/*z* 355 [*M*+H]^+^, *t*_R_=3.3–3.4 min; HRMS (ESI) calcd for C_23_H_35_N_2_O 355.2744 [*M*+H]^+^, found 355.2733.

**2-(3,4-Dichlorophenyl)-2-methyl-*N*-[*trans*-(±)-2-(pyrrolidin-1-yl)cyclohexyl]propanamide (56):** Prepared according to Method C from *trans*-(±)-*N*-methyl-2-(pyrrolidin-1-yl)cyclohexanamine (91 mg, 0.5 mmol), 4-chloro-α,α-dimethylacetic acid (99 mg, 0.5 mmol), Et_3_N (0.2 mL), and PyBrop (312 mg, 0.6 mmol) in CH_2_Cl_2_ (anhydrous, 1 mL). The crude residue was purified by column chromatography eluting with CH_2_Cl_2_/MeOH/NH_3_ 95:5:0.1 to afford **56** (121 mg, 67 %). ^1^H NMR (500 MHz, [D_6_]DMSO): *δ*=8.91 (bs, 1H0, 7.43 (d, 2 H, *J*=8.4 Hz, ArH), 7.21 (d, 2 H, *J*=8.4 Hz, ArH), 4.59 (bs, 1 H), 3.89 (bs, 1 H), 3.31 (bs, 1 H), 3.16–3.08 (m, 2 H), 2.28 (s, 3 H, CH_3_), 2.11 (d, 1 H, *J*=10.2 Hz), 1.87–1.82 (m, 3 H), 1.78–1.76 (m, 1 H), 1.72 (d, 1 H, *J*=12.2 Hz), 1.66 (d, 1 H, *J*=12.2 Hz), 1.55 (s, 3 H, CH_3_), 1.50–1.43 (m, 3 H), 1.37 (s, 3 H, CH_3_), 1.28–1.26 (m, 1 H), 1.20–1.14 ppm (m, 1 H); ^13^C NMR (125 MHz, [D_6_]DMSO): *δ*=175.8, 144.3, 131.0, 128.8, 126.8, 59.8, 50.8, 50.0, 46.8, 31.2, 30.5, 28.0, 26.0, 25.2, 23.9, 23.5, 23.0, 22.5 ppm; LC–MS: *m*/*z* 363 and 365 ^35^Cl and ^37^Cl, [*M*+H]^+^, *t*_R_=3.2–3.4 min; HRMS (ESI) calcd for C_21_H_32_ClN_2_O 363.2198 ^35^Cl [*M*+H]^+^, found 363.2180.

**1-(3,4-Dichlorophenyl)-*N*-[*trans*-(±)-2-(pyrrolidin-1-yl)cyclohexyl]cyclopropanecarboxamide (57):** Prepared according to Method C from *trans*-(±)-*N*-methyl-2-(pyrrolidin-1-yl)cyclohexanamine (91 mg, 0.5 mmol), 1-(3,4-dichlorophenyl)cyclopropanecarboxylic acid (115 mg, 0.5 mmol), Et_3_N (0.2 mL), and PyBrop (312 mg, 0.6 mmol) in CH_2_Cl_2_ (anhydrous, 1 mL). The crude residue was purified by column chromatography eluting with CH_2_Cl_2_/MeOH/NH_3_ 95:5:0.1 to afford **57** (33 mg, 17 %). ^1^H NMR (500 MHz, CDCl_3_): *δ*=7.41 (d, 1 H, ArH, *J*=8.3 Hz), 7.29 (d, 1 H, ArH, *J*=2.1 Hz), 7.03 (dd, 1 H, ArH, *J*=8.3 and 2.1 Hz), 4.51 (bs, 1 H), 3.91 (bs, 1 H), 3.75 (bs, 1 H), 3.54 (bs, 2 H), 3.25 (bs, 1 H), 3.15 (bs, 1 H), 2.75 (s, 3 H, CH_3_), 2.26–2.18 (m, 2 H), 2.13–2.01 (m, 4 H), 1.72–1.69 (m, 4 H), 1.63–1.58 (m, 3 H), 1.45–1.42 (m, 2 H), 1.40–1.39 (m, 1 H), 0.85 ppm (bs, 1 H); ^13^C NMR (125 MHz, [D_6_]DMSO): *δ*=171.4, 141.8, 131.9, 130.8, 130.7, 119.0, 109.2, 60.1, 51.5, 48.4, 40.2, 28.6, 27.5, 24.1, 23.9, 23.8, 23.6, 23.4, 23.1 ppm; LC–MS: *m*/*z* 395 and 397 ^35^Cl and ^37^Cl [*M*+H]^+^, *t*_R_=3.1–3.3 min; HRMS (ESI) calcd for C_21_H_29_Cl_2_N_2_O 395.1651 ^35^Cl [*M*+H]^+^, found 395.1634.

## References

[1] Stuart K, Brun R, Croft SL, Fairlamb AH, Gurtler RE, McKerrow J, Reed S, Tarleton R (2008). J. Clin. Invest..

[2a] Renslo AR, McKerrow JH (2006). Nat. Chem. Biol..

[2b] Pepin J, Milord F (1994). Adv. Parasitol..

[2c] Priotto G, Kasparian S, Mutombo W, Ngouama D, Ghorashian S, Arnold U, Ghabri S, Baudin E, Buard V, Kazadi-Kyanza S, Ilunga M, Mutangala W, Pohlig G, Schmid C, Karunakara U, Torreele E, Kande V (2009). Lancet.

[2d] Thuita JK, Karanja SM, Wenzler T, Mdachi RE, Ngotho JM, Kagira JM, Tidwell R, Brun R (2008). Acta. Trop..

[3] Barrett MP, Boykin DW, Brun R, Tidwell R (2007). Br. J. Pharmacol..

[4] Jones DC, Hallyburton I, Stojanovski L, Read KD, Frearson JA, Fairlamb AH (2010). Biochem. Pharmacol..

[5a] Szmuszkovicz J, VonVoigtlander PF (1982). J. Med. Chem..

[5b] de Costa BR, Bowen WD, Hellewell SB, George C, Rothman RB, Reid AA, Walker JM, Jacobson AE, Rice KC (1989). J. Med. Chem..

[6] Gonzalez-Sabin J, Gotor V, Rebolledo F (2004). Chem. Eur. J..

[7] Clark CR, Halfpenny PR, Hill RG, Horwell DC, Hughes J, Jarvis TC, Rees DC, Schofield D (1988). J. Med. Chem..

[8] Renner S, Schneider G (2006). ChemMedChem.

[9a] Hawkins PCD, Skillman AG, Nicholls A (2007). J. Med. Chem..

[9b] Nicholls A, McGaughey GB, Sheridan RP, Good AC, Warren G, Mathieu M, Muchmore SW, Brown SP, Grant JA, Haigh JA, Nevins N, Jain AN, Kelley B (2010). J. Med. Chem..

