# Supplementary material for: Optimisation of the Anti-Trypanosoma brucei Activity of the Opioid Agonist U50488
Source: ChemMedChem. 2011 Aug 10;6(10):1832–40. doi: 10.1002/cmdc.201100278 (PMC3229842; doi:10.1002/cmdc.201100278)
Supplement: Supplementary file 1 [file cmdc0006-1832-SD1.pdf]

CHEMMEDCHEM
Chemistry & Drug Discovery

Supporting Information

© Copyright Wiley-VCH Verlag GmbH & Co. KGaA, 69451 Weinheim, 2011

Optimisation of the Anti-*Trypanosoma brucei* Activity of the Opioid Agonist U50488

Victoria C. Smith, Laura A. T. Cleghorn, Andrew Woodland, Daniel Spinks, Irene Hallyburton, Iain T. Collie, N. Yi Mok, Suzanne Norval, Ruth Brenk, Alan H. Fairlamb, Julie A. Frearson, Kevin D. Read, Ian H. Gilbert,* and Paul G. Wyatt*[a]

cmdc_201100278_sm_miscellaneous_information.pdf

Supplementary Information

***trans*-(±)-*N*-Ethyl-2-(pyrrolidin-1-yl)cyclohexanamine 5a**
Prepared according to the procedure for **5** and **7**, from ethyl amine on a 6 mmol scale. An inseparable 2:1 mixture of (1R,2R)-N-ethyl-2-(pyrrolidin-1-yl)cyclohexanamine and (1R,2R)-N-(2-methoxyethyl)-2-(pyrrolidin-1-yl)cyclohexanamine was produced which was used without further purification.

***trans*-(±)-*N*-(tert-butyl)-2-(pyrrolidin-1-yl)cyclohexanamine 5b**
Prepared according to method D, from *t*-butyl amine on a 5.9 mmol scale to afford (1R,2R)-N-(tert-butyl)-2-(pyrrolidin-1-yl)cyclohexanamine as a yellow oil (0.25 g, 1.1 mmol, 19 % yield). ^{1}H NMR (500 MHz, $CDCl_3$) δ 2.45-2.29 (4H, m), 2.23 (1H, bs), 1.96-1.94 (1H, m), 1.67-1.47 (8H, m), 1.11-1.02 (5H, m), 0.98 (9H, s).

***trans*-(±)-*N*-Benzyl-2-(pyrrolidin-1-yl)cyclohexanamine 5c**
Prepared according to method D, from benzylamine on a 5.9 mmol scale to afford (1R,2R)-N-benzyl-2-(pyrrolidin-1-yl)cyclohexanamine as a yellow oil (1.08 g, 4.2 mmol, 71% yield). ^{1}H NMR (500 MHz, $CDCl_3$) δ 7.27-7.15 (5H, m), 3.84 (1H, d, *J* 13.3 *Hz*), 3.55 (1H, d, *J* 13.3 *Hz*), 2.98 (bs), 2.44-2.32 (4H, m), 2.23 (1H, td, *J* 10.2 and 4.0 *Hz*), 2.07-2.04 (1H, m), 1.70-1.51 (8H, m), 1.31-0.99 (4H, m).

***trans*-(±)-2-(Pyrrolidin-1-yl)cyclohexanamine 5d.** Methanesulfonyl Chloride (1.72 mL, 22 mmol) was added dropwise to an ice-cold solution of ***trans*-(±)-2-(pyrrolidin-1-yl)cyclohexanol (1)** (20 mmol) and triethylamine (5.6 mL, 40 mmol) in anhydrous diethylether (50 mL). A creamy white precipitate formed and the reaction mixture was stirred for 30 min, then a further (2.8 mL, 20 mmol) triethylamine was added. The reaction was removed from the cooling bath and ammonia (36% aqueous solution, 48 mL) was added. The resulting 2 phase reaction mixture was allowed to warm to rt and stirred vigorously for 16 h. The layers were separated, the aqueous layer further extracted with diethylether (80 mL) and the combined organics dried over $MgSO_4$ and concentrated *in vacuo*. The title compound was obtained as a colourless gum (90%). ^{1}H NMR (CDCl3) δ 2.54-2.60 (m, 2H), 2.49-2.54 (m, 2H), 2.23 (m, 1H), 2.13-2.19 (m, 1H), 1.86-1.94 (m, 2H), 1.76-1.82 (m, 2H), 1.61-1.68 (m, 4H), 1.02-1.15 (m, 4H). LCMS *m/z*: 169 $[M+H]^+$, T_R: 0.6-0.8 min.

***trans*-(±)-4-(Pyrrolidin-1-yl)tetrahydrofuran-3-ol 5e**
3,4-Epoxytetrahydrofuran (5.0 g, 5.8 mmol) and pyrrolidine (5.0 mL, 6.0 mmol) were dissolved in ^{i}PrOH (20 mL), heated at 80ºC for 20 h, cooled to room temperature and concentrate *in-vacuo* to give (3S,4R)-4-(pyrrolidin-1-yl)tetrahydrofuran-3-ol as a brown solid (8.8 g, 5.6 mmol, 96 % yield). ^{1}H NMR (500 MHz, $CDCl_3$) δ 4.37 (1H, quint, *J* = 2.6 Hz), 4.09 (1H, dd, *J* = 9.1 and 6.5 Hz), 3.98 (1H, dd, *J* = 9.9 and 5.2 Hz), 3.75 (1H, dd, *J* = 9.9 and 2.6 Hz), 3.69 (1H, *J* = 9.2 and 6.2 Hz), 2.77 (1H, td, *J* = 6.2 and 2.6 Hz), 2.62-2.45 (4H, m), 2.24 (1H, bs), 1.86-1.75 (4H, m).

***trans*-(±)-2-(Pyrrolidin-1-yl)cyclopentanol 5f**
Cyclopentene oxide (5.0 g, 58 mmol) and pyrrolidine (5.0 mL, 60 mmol) were added to ^{i}PrOH (20 mL) and the reaction stirred at 80ºC for 24 h then cooled to room temperature and concentrated under reduced pressure to give (1R,2R)-2-(pyrrolidin-1-yl)cyclopentanol as a brown solid (8.1 g, 52 mmol, 90 % yield). ^{1}H NMR (500 MHz,

$CDCl_3$) δ 4.17 (1H, dd, *J* = 12.2 and 5.1 Hz), 2.65-2.59 (4H, m), 2.45 (1H, td, *J* = 8.0 and 5.1 Hz), 2.02-1.93 (3H, m),1.84-1.52 (8H, m).

***trans*(±)-*N*-Methyl-4-(pyrrolidin-1-yl)tetrahydrofuran-3-amine 8c**
Triethylamine (4.17 mL, 30 mmol) was added to a solution of (3S,4R)-4-(pyrrolidin-1-yl)tetrahydrofuran-3-ol (1.57 g, 10 mmol) in Et_2O (15 mL) cooled to 0°C. MsCl (0.93 mL, 12 mmol) was added dropwise and the solution was stirred for 10 min then warmed to rt, methylamine (1.51 mL, 20 mmol) was added and the reaction stirred for a further 16 h, diluted with ether and washed with H_2O, the organic layer was concentrated *in-vacuo*. Column chromatography eluting with EtOAc: MeOH 95:5 afforded the desired di-amine as a bright yellow oil (0.64g, 38 %). (^{1}H NMR, $CDCl_3$), δ 4.14-4.11 (m, 1H), 4.05 (dd, 1H, *J* = 10.2 and 5.9 Hz), 3.94 (dd, 1H, *J* = 9.2 and 6.5 Hz), 3.74 (dd, 1H, *J* = 10.2 and 3.7 Hz), 3.59 (dd, 1H, *J* = 9.2 and 5.4 Hz), 2.92 (td, 1H, *J* = 6.0 and 3.3 Hz), 2.45-2.41 (m, 4H, 2 x pyrrolidine CH_2), 1.66-1.59 (m, 4H, 2 x pyrrolidine CH_2), 1.41 (s, 3H, CH_3).

***trans*(±)-*N*-Methyl-2-(pyrrolidin-1-yl)cyclopentanamine 8d**
Triethylamine (4.17 mL, 30 mmol) was added to a solution of trans(±)-*N*-methyl-2-(pyrrolidin-1-yl)cyclopentanamine (1.53 g, 10 mmol) in Et_2O (15 mL) cooled to 0 °C. MsCl (0.93 mL, 12 mmol) was added dropwise and the solution was stirred for 10 min then warmed to rt, methylamine (1.51 mL, 20 mmol) was added and the reaction stirred for a further 16 h, diluted with ether and washed with H_2O, the organic layer was concentrated *in-vacuo*. Column chromatography eluting with EtOAc: MeOH 95:5 afforded the desired di-amine as a yellow oil (0.68, 40 %). (^{1}H NMR, $CDCl_3$), δ 4.12-4.09 (m, 1H), 2.74-2.70 (m, 1H), 2.53-2.50 (m, 4H), 2.18-2.11 (m, 1H), 1.94-1.92 (m, 1H), 1.88-1.82 (m, 1H), 1.71-1.67 (m, 5H), 1.65-1.61 (m, 1H), 1.51-1.49 (m, 4H).

***N*-Methyl-2-phenyl-*N*-(*trans*-(±))-(2-(pyrrolidin-1-yl)cyclohexyl)acetamide 10**
Prepared according to method B from *trans*-(±)-*N*-methyl-2-(pyrrolidin-1-yl)cyclohexanamine (100 mg, 0.55 mmol) and phenylacetyl chloride (93 mg, 0.6 mmol) in CH_2Cl_2 (anhydrous, 5 mL) in 91 % yield. ^{1}H NMR (*d6*-DMSO) δ 9.59 (br s, HCl), 7.28-7.35 (m, 2H, ArH), 7.20-7.26 (m, 3H, ArH), 4.51-4.62 (m, 1H), 3.82 (d, 1H, *J* = 15.9 Hz), 3.75 (d, 1H, *J* = 15.9 Hz), 3.54-3.62 (m, 1H), 3.39-3.47 (m, 1H), 3.15-3.28 (m, 3H), 2.92 (s, 3H, CH_3), 2.05-2.12 (m, 1H), 1.80-1.95 (m, 4H), 1.73-1.80 (m, 1H), 1.66-1.72 (m, 1H), 1.47-1.61 (m, 3H), 1.17-1.37 (m, 2H). LCMS *m/z*: 301 $[M+H]^+$, T_R: 3.9 min. HRMS (ESI) calcd. for $C_{19}H_{29}N_2O$ 301.2280 $[M+H]^+$, found 301.2287.

2-(3-Chlorophenyl)-*N*-methyl-*N*-(*trans*-(±))-(2-(pyrrolidin-1-yl)cyclohexyl)acetamide.HCl 11
Prepared according to method A from *trans*-(±)-*N*-methyl-2-(pyrrolidin-1-yl)cyclohexanamine (100 mg, 0.55 mmol), 3-chlorophenylacetic acid (188 mg, 1.1 mmol), HOBt (149 mg, 1.1 mmol), DIPEA (192 μL, 1.1 mmol) and EDCI (211 mg, 1.1 mmol) in DMF (anhydrous, 5 mL) in 57 % yield. ^{1}H NMR (*d6*-DMSO) δ 9.83 (br s, HCl), 7.31-7.35 (m, 2H, ArH), 7.27-7.30 (m, 1H, ArH), 7.19-7.23 (m, 1H, ArH), 4.48-4.62 (m, 1H), 3.95 (d, 1H, *J* = 16.2 Hz), 3.73 (d, 1H, *J* = 16.2 Hz), 3.52-3.64 (m, 1H), 3.42-3.50 (m, 1H), 3.12-3.28 (m, 3H), 2.96 (s, 3H, CH_3), 2.03-2.11 (m, 1H), 1.81-1.98 (m, 4H), 1.73-1.80 (m, 1H), 1.66-1.73 (m, 1H), 1.47-1.62 (m, 3H), 1.21-1.38 (m, 2H).

LCMS *m/z*: 335 [M+H]$^+$, T_R: 3.6 min. HRMS (ESI) calculated for $C_{19}H_{28}N_2OCl$ 335.1890 [M+H]$^+$, found 335.1899.

2-(4-Chlorophenyl)-*N*-methyl-*N*-(*trans*-(±))-(2-(pyrrolidin-1-yl)cyclohexyl)acetamide.HCl 12

Prepared according to method B from *trans*-(±)-*N*-methyl-2-(pyrrolidin-1-yl)cyclohexanamine (100 mg, 0.55 mmol) and 4-chlorophenylacetyl chloride (88 μL, 0.6 mmol) in CH_2Cl_2 (anhydrous, 5 mL) in 74 % yield. ^{1}H NMR (*d6*-DMSO) δ 9.27 (br s, HCl), 7.36 (d, 2H, ArH), 7.25 (d, 2H, ArH), 4.47-4.61 (m, 1H), 3.80 (d, 1H, *J* = 16.2 Hz), 3.73 (d, 1H, *J* = 16.2 Hz), 3.54-3.62 (m, 1H), 3.36-3.43 (m, 1H), 3.15-3.28 (m, 3H), 2.91 (s, 3H, CH_3), 2.03-2.10 (m, 1H), 1.81-1.92 (m, 4H), 1.73-1.79 (m, 1H), 1.65-1.71 (m, 1H), 1.51-1.60 (m, 3H), 1.21-1.29 (m, 2H). LCMS *m/z*: 335 [M+H]$^+$, T_R: 3.6 min. HRMS (ESI) calculated for $C_{19}H_{28}N_2OCl$ 335.1890 [M+H]$^+$, found 335.1869.

2-(2,6-Dichlorophenyl)-*N*-methyl-*N*-(*trans*-(±))-(2-(pyrrolidin-1-yl)cyclohexyl)acetamide.HCl 13

Prepared according to method A from *trans*-(±)-*N*-methyl-2-(pyrrolidin-1-yl)cyclohexanamine (100 mg, 0.55 mmol), 2.6-dichlorophenylacetic acid (226 mg, 1.1 mmol), HOBt (149 mg, 1.1 mmol), DIPEA (192 μL, 1.1 mmol) and EDCI (211 mg, 1.1 mmol) in DMF (anhydrous, 5 mL) in 48% yield. ^{1}H NMR ($CDCl_3$) δ11.53 (br s, HCl), 7.31 (d, 2H, ArH, *J* = 8.1 Hz), 7.14 (t, 1H, ArH, *J* = 8.1 Hz), 4.56 (br s, 1H), 4.38 (br d, 1H, *J* = 16.7 Hz), 3.99 (d, 1H, *J* = 16.7 Hz), 3.85-3.93 (m, 1H), 3.61-3.68 (m, 1H), 3.54 (br s, 1H), 3.28 (s, 3H, CH_3), 3.06-3.14 (m, 1H), 2.86-2.96 (m, 1H), 2.12-2.31 (m, 3H), 1.81-1.94 (m, 6H), 1.45-1.55 (m, 1H), 1.23-1.40 (m, 2H). LCMS *m/z*: 369 [M+H]$^+$, T_R: 3.7 min. HRMS (ESI) calcd. for $C_{19}H_{27}N_2OCl_2$ 369.1500 [M+H]$^+$, found 369.1502.

2-(2,4-Dichlorophenyl)-*N*-methyl-*N*-(*trans*-(±))-(2-(pyrrolidin-1-yl)cyclohexyl)acetamide.HCl 14

Prepared according to method A from *trans*-(±)-*N*-methyl-2-(pyrrolidin-1-yl)cyclohexanamine (100 mg, 0.55 mmol), 2,4-dichlorophenylacetic acid (226 mg, 1.1 mmol), HOBt (149 mg, 1.1 mmol), DIPEA (192 μL, 1.1 mmol) and EDCI (211 mg, 1.1 mmol) in DMF (anhydrous, 5 mL) in 39 % yield. ^{1}H NMR (*d6*-DMSO) δ 9.53 (br s, HCl), 7.57-7.60 (m, 1H, ArH), 7.38-7.41 (m, 2H, ArH), 4.48-4.60 (m, 1H), 3.94 (d, 1H, *J* = 16.6 Hz), 3.87 (d, 1H, *J* = 16.6 Hz), 1.57-1.65 (m, 1H), 3.41-3.48 (m, 1H), 3.22-3.29 (m, 1H), 3.13-3.21 (m, 2H), 2.99 (s, 3H, CH_3), 2.05-2.11 (m, 1H), 1.81-1.95 (m, 4H), 1.74-1.80 (m, 1H), 1.67-1.73 (m, 1H), 1.47-1.65 (m, 3H),1.21-1.36 (m, 2H). LCMS *m/z*: 369 [M+H]$^+$, T_R: 3.8 min. HRMS (ESI) calcd. for $C_{19}H_{27}N_2OCl_2$ 369.1500 [M+H]$^+$, found 369.1477.

2-(3,4-Difluorophenyl)-*N*-methyl-*N*-(*trans*-(±))-(2-(pyrrolidin-1-yl)cyclohexyl)acetamide.HCl 15

Prepared according to method A from *trans*-(±)-*N*-methyl-2-(pyrrolidin-1-yl)cyclohexanamine (100 mg, 0.55 mmol), 3,4-difluorophenylacetic acid (189 mg, 1.1 mmol), HOBt (149 mg, 1.1 mmol), DIPEA (192 μL, 1.1 mmol) and EDCI (211 mg, 1.1 mmol) in DMF (anhydrous, 5 mL) in 52 % yield. ^{1}H NMR (*d6*-DMSO) δ 9.65 (br s, HCl), 7.28-7.37 (m, 2H, ArH), 7.05-7.10 (m, 1H, ArH), 4.47-4.60 (m, 1H), 3.88-3.96 (m, 1H), 3.66-3.73 (m, 1H), 3.52-3.61 (m, 1H), 3.41-3.49 (m, 1H), 3.11-3.28 (m, 3H), 2.94 (s, 3H, CH_3), 2.03-2.10 (m, 1H), 1.80-1.97 (m, 4H), 1.72-1.79 (m, 1H), 1.65-1.72 (m, 1H), 1.46-1.64 (m, 3H), 1.19-1.36 (m, 2H). LCMS *m/z*: 337 [M+H]$^+$, T_R: 3.3 min.

2-(3-Fluorophenyl)-*N*-methyl-*N*-(*trans*-(±))-(2-(pyrrolidin-1-yl)cyclohexyl)acetamide.HCl 16

Prepared according to method A from *trans*-(±)-*N*-methyl-2-(pyrrolidin-1-yl)cyclohexanamine (100 mg, 0.55 mmol), 2-(3-fluorophenyl)acetic acid (169 mg, 1.1 mmol), HOBt (149 mg, 1.1 mmol), DIPEA (192 μL, 1.1 mmol) and EDCI (211 mg, 1.1 mmol) in DMF (anhydrous, 5 mL) in 43 % yield. ^1H NMR ($CDCl_3$) δ 11.33 (br s, 1H, HCl), 7.23-7.28 (m, 1H, ArH), 7.09 (d, 1H, ArH, *J* = 7.6 Hz), 7.02 (d, 1H, ArH, *J* = 10.0 Hz), 6.92 (td, 1H, ArH, *J* = 8.4 and 2.2 Hz), 4.66-4.83 (m, 1H), 4.15 (d, 1H *J* = 15.6 Hz), 3.89-3.99 (m, 1H), 3.83 (d, 1H, *J* = 15.6 Hz), 3.56-3.65 (m, 1H), 3.22-3.36 (m, 1H), 3.08 (s, 3H, CH_3), 2.97-3.10 (m, 2H), 2.15-2.32 (m, 2H), 2.07-2.14 (m, 1H), 1.92-1.98 (m, 1H), 1.82-1.92 (m, 3H), 1.75-1.82 (m, 1H), 1.54-1.66 (m, 1H), 1.44-1.53 (m, 1H), 1.24-1.44 (m, 2H). LCMS *m/z*: 319 $[M+H]^+$, T_R: 3.5 min. HRMS (ESI) calcd. for $C_{19}H_{28}N_2OF$ 319.2186 $[M+H]^+$, found 319.2169.

2-(4-Fluorophenyl)-*N*-methyl-*N*-(*trans*-(±))-(2-(pyrrolidin-1-yl)cyclohexyl)acetamide.HCl 17

Prepared according to method B from *trans*-(±)-*N*-methyl-2-(pyrrolidin-1-yl)cyclohexanamine (100 mg, 0.55 mmol) and 2-(4-fluorophenyl)acetyl chloride (83 μL, 0.6 mmol) in CH_2Cl_2 (anhydrous, 5 mL) in 30 % yield. ^1H NMR ($CDCl_3$) δ 11.37 (br s, 1H, HCl), 7.28 (dd, 2H, ArH, *J* = 8.7 and 5.5 Hz), 6.98 (t, 2H, ArH, *J* = 8.7 Hz), 4.64-4.82 (m, 1H), 4.16 (d, 1H, *J* = 16.3 Hz), 3.90-3.99 (m, 1H), 3.75 (d, 1H, *J* = 16.3 Hz), 3.56-3.63 (m, 1H), 3.22-3.34 (m, 1H), 3.09 (s, 3H, CH_3), 2.96-3.06 (m, 2H), 2.23-2.31 (m, 1H), 2.14-2.22 (m, 1H), 2.07-2.13 (m, 1H), 1.92-1.98 (m, 1H), 1.81-1.90 (m, 3H), 1.75-1.81 (m, 1H), 1.54-1.64 (m, 1H), 1.43-1.52 (m, 1H), 1.24-1.42 (m, 2H). LCMS *m/z*: 319 $[M+H]^+$, T_R: 3.3 min. HRMS (ESI) calcd. for $C_{19}H_{28}N_2OF$ 319.2186 $[M+H]^+$, found 319.2182.

2-(3-Methoxyphenyl)-*N*-methyl-*N*-(*trans*-(±))-(2-(pyrrolidin-1-yl)cyclohexyl)acetamide.HCl 18

Prepared according to method B from *trans*-(±)-*N*-methyl-2-(pyrrolidin-1-yl)cyclohexanamine (100 mg, 0.55 mmol) and 2-(3-methoxyphenyl)acetyl chloride (94 μL, 0.6 mmol) inCH_2Cl_2 (anhydrous, 5 mL) in 19 % yield. ^1H NMR (*d6*-DMSO) δ 9.52 (br s, 1H, HCl), 7.19-7.25 (m, 1H, ArH), 6.78-6.85 (m, 3H, ArH), 4.51-4.62 (m, 1H), 3.74-3.76 (m, 2H), 3.74 (s, 3H, Ph-OCH_3), 3.54-3.62 (m, 1H), 3.39-3.46 (m, 1H), 3.16-3.26 (m, 3H), 2.91 (s, 3H, CH_3), 2.05-2.12 (m, 1H), 1.81-1.94 (m, 4H), 1.73-1.80 (m, 1H), 1.66-1.72 (m, 1H), 1.47-1.60 (m, 3H), 1.22-1.35 (m, 2H). LCMS *m/z*: 331 $[M+H]^+$, T_R: 3.3 min. HRMS (ESI) calcd. for $C_{20}H_{31}N_2O_2$ 331.2386 $[M+H]^+$, found 331.2395.

2-(4-Methoxyphenyl)-*N*-methyl-*N*-(*trans*-(±))-(2-(pyrrolidin-1-yl)cyclohexyl)acetamide.HCl 19

Prepared according to method B from *trans*-(±)-*N*-methyl-2-(pyrrolidin-1-yl)cyclohexanamine (100 mg, 0.55 mmol) and 2-(4-methoxyphenyl)acetyl chloride (92 μL, 0.6 mmol) in CH_2Cl_2 (anhydrous, 5 mL) in 79 % yield. ^1H NMR ($CDCl_3$) δ 11.40 (br s, 1H, HCl), 7.24 (d, 2H, ArH, *J* = 8.65 Hz), 6.87 (d, 2H, ArH, *J* = 8.65 Hz), 4.65-4.85 (m, 1H), 4.03 (d, 1H, *J* = 15.8 Hz), 3.87-3.97 (m, 1H), 3.81 (d, 1H, *J* = 15.8 Hz), 3.81 (s, 3H, Ph-OCH_3), 3.60-3.69 (m, 1H), 3.26-3.41 (m, 1H), 3.09 (s, 3H, CH_3), 2.94-3.09 (m, 2H), 2.12-2.32 (m, 3H), 1.92-1.99 (m, 1H), 1.82-1.92 (m, 3H), 1.75-1.81 (m, 1H), 1.58-1.68 (m, 1H), 1.46-1.55 (m, 1H), 1.25-1.45 (m, 2H). LCMS *m/z*:

331 [M+H]$^+$, T_R: 3.4 min. HRMS (ESI) calcd. for $C_{20}H_{31}N_2O_2$ 331.2386 [M+H]$^+$, found 331.2389.

***N*-Methyl-*N*-(*trans*-(±))-2-(pyrrolidin-1-yl)cyclohexyl)-2-(o-tolyl)acetamide.HCl 20**

Prepared according to method A from *trans*-(±)-*N*-methyl-2-(pyrrolidin-1-yl)cyclohexanamine (100 mg, 0.55 mmol), 2-methylphenylacetic acid (165 mg, 1.1 mmol), HOBt (149 mg, 1.1 mmol), DIPEA (192 μL, 1.1 mmol) and EDCI (211 mg, 1.1 mmol) in DMF (anhydrous, 5 mL) in 52 % yield. ^{1}H NMR (*d6*-DMSO) δ 7.59 (br s, 1H, HCl), 7.07-7.18 (m, 4H, ArH), 4.52-4.63 (m, 1H), 3.75-3.78 (m, 2H), 3.56-3.65 (m, 1H), 3.37-3.48 (m, 2H), 3.22-3.29 (m, 1H), 3.15-3.22 (m, 2H), 2.95 (s, 3H, CH_3), 2.18-2.23 (m, 3H, Ph-CH_3), 2.06-2.12 (m, 1H), 1.81-1.97 (m, 4H), 1.75-1.81 (m, 1H), 1.68-1.74 (m, 1H), 1.48-1.64 (m, 3H), 1.20-1.39 (m, 2H). LCMS *m/z*: 315 [M+H]$^+$, T_R: 3.3 min. HRMS (ESI) calcd. for $C_{20}H_{31}N_2O$ 315.2436 [M+H]$^+$, found 315.2431.

***N*-Methyl-*N*-(*trans*-(±))-(2-(pyrrolidin-1-yl)cyclohexyl)-2-(m-tolyl)acetamide.HCl 21**

Prepared according to method A from *trans*-(±)-*N*-methyl-2-(pyrrolidin-1-yl)cyclohexanamine (100 mg, 0.55 mmol), 2-(*m*-tolyl)acetic acid (165 mg, 1.1 mmol), HOBt (149 mg, 1.1 mmol), DIPEA (192 μL, 1.1 mmol) and EDCI (211 mg, 1.1 mmol) in DMF (anhydrous, 5 mL) in 29 % yield. ^{1}H NMR ($CDCl_3$) δ 11.40 (br s, 1H, HCl), 7.19 (t, 1H, ArH, *J* = 7.6 Hz), 7.10 (s, 1H, ArH), 7.08 (d, 1H, ArH, *J* = 7.6 Hz), 7.04 (d, 1H, ArH, *J* = 7.6 Hz), 4.63-4.83 (m, 1H), 3.99 (d, 1H, *J* = 15.6 Hz), 3.85-3.94 (m, 1H), 3.85 (d, 1H, *J* = 15.6 Hz), 3.59-3.67 (m, 1H), 3.26-3.39 (m, 1H), 3.06 (s, 3H, CH_3), 3.00-3.07 (m, 1H), 2.91-3.00 (m, 1H), 2.32 (s, 3H, Ph-CH_3), 2.11-2.27 (m, 3H), 1.90-1.97 (m, 1H), 1.80-1.89 (m, 3H), 1.73-1.79 (m, 1H), 1.55-1.66 (m, 1H), 1.44-1.53 (m, 1H), 1.24-1.43 (m, 2H). LCMS *m/z*: 315 [M+H]$^+$, T_R: 3.5 min. HRMS (ESI) calcd. for $C_{20}H_{31}N_2O$ 315.2436 [M+H]$^+$, found 315.2432.

***N*-Methyl-*N*-((trans±)-2-(pyrrolidin-1-yl)cyclohexyl)-2-(*p*-tolyl)acetamide 22**

Prepared according to method C from *trans*-(±)-*N*-ethyl-2-(pyrrolidin-1-yl)cyclopentanamine (130 mg, 0.5 mmol), 4-tolylacetic acid (75 mg, 0.5 mmol), triethylamine (0.2 mL) and PyBrop (312 mg, 0.6 mmol) in CH_2Cl_2 (anhydrous, 1 mL). The crude residue was purified by column chromatography eluting withCH_2Cl_2 :MeOH:NH_3 95:5:0.1 to afford **22** (48 mg, 31 %).^{1}H NMR (DMSO) δ = 8.82 (bs, 1H), 7.13 (d, 4H, *J* = 2.1 Hz, ArH), 4.56 (bs, 1H), 3.74 (d, 1H, *J* = 15.7 Hz, CH_2), 3.64 (d, 1H, *J* = 15.7 Hz, CH_2), 3.61-3.57 (m, 1H), 3.32 (s, 3H, CH_3), 3.27-3.22 (m, 3H), 2.87 (s, 3H, CH_3), 2.29 (s, 3H), 2.11 (s, 2H), 1.92-1.87 (m, 3H), 1.83-1.79 (m, 1H), 1.57-1.52 (m, 3H), 1.33-1.23 (m, 2H). LCMS: *m/z* 315 [M+H]$^+$, T_R = 1.6-1.9 min. HRMS (ESI) calculated for $C_{20}H_{31}N_2O$ 315.2431 [M+H]$^+$found 315.2420.

2-(4-Isopropylphenyl)-*N*-methyl-*N*-((trans(±)-2-(pyrrolidin-1-yl)cyclohexyl)acetamide 23

Prepared according to method C from *trans*-(±)-*N*-ethyl-2-(pyrrolidin-1-yl)cyclopentanamine (130 mg, 0.5 mmol), 4-isopropylphenylacetic acid (89 mg, 0.5 mmol), triethylamine (0.2 mL) and PyBrop (312 mg, 0.6 mmol) inCH_2Cl_2 (anhydrous, 1 mL). The crude residue was purified by column chromatography eluting with CH_2Cl_2 : MeOH:NH_3 95:5:0.1 to afford **23** (58 mg, 34 %).^{1}H NMR (DMSO) δ = 8.83 (bs, 1H), 7.19 (d, 1H, *J* = 8.2 Hz, ArH), 7.14 (d, 1H, *J* = 8.2 Hz, ArH), 4.56 (bs, 1H), 3.73 (d, 1H, *J* = 15.8 Hz, CH_2), 3.65 (d, 1H, *J* = 15.8, CH2), 3.61-3.59 (m, 1H), 3.23-

3.19 (m, 2H), 2.88 (s, 3H, CH_3), LCMS: *m/z* 343[M+H]$^+$, T_R = 3.2-3.3 min. HRMS (ESI) calculated for $C_{22}H_{35}N_2O$ 343.2744 [M+H]$^+$found 343.2736.

trans(±)-*N*-Methyl-2-(pyrrolidin-1-yl)-*N*-(3-trifluoromethyl)phenethyl)cyclohexanamine 24

Prepared according to method C from *trans*-(±)-*N*-methyl-2-(pyrrolidin-1-yl)cyclohexanamine (91 mg, 0.5 mmol), 3-(trifluoromethyl)phenyl acetic acid (102 mg, 0.5 mmol), triethylamine (0.2 mL) and PyBrop (312 mg, 0.6 mmol) in CH_2Cl_2 (anhydrous, 1 mL). The crude residue was purified by column chromatography eluting with CH_2Cl_2 to CH_2Cl_2 : MeOH 95:5 to afford **24** (92 mg, 50 %). (^{1}H NMR, d^6 DMSO) δ = 8.84 (bs, 1H), 7.43-7.56 (m, 4H), 6.96 (bs, 1H), 4.72 (bs, 1H), 4.12 (d, 1H, *J* = 16.7 Hz), 3.97 (bs, 1H), 3.80 d, 1H, *J* = 16.7 Hz), 3.65 (bs, 1H), 3.47-3.54 (m, 2H), 3.24 (dd, 2H, *J* = 14.7 and 7.4 Hz), 3.15-3.18 (m, 2H), 3.02 (s, 3H, CH_3), 2.27 (m, 1H), 2.13-2.16 (m, 2H), 1.98-2.00 (m, 3H), 1.89-1.92 (m, 1H), 1.82-1.85 (m, 2H), 1.48-1.53 (m, 1H). LCMS: *m/z* 369 [M+H]$^+$, T_R = 3.1-3.2 min. HRMS: (ESI) calculated for $C_{20}H_{28}F_3N_2O$ 343.2744 [M+H]$^+$found 343.2736.

***N*-((trans(±))-2-(Pyrrolidin-1-yl)cyclohexyl)-2-(4-(trifluoromethyl)phenyl)acetamide 25**

Prepared according to method C from *trans*-(±)-*N*-methyl-2-(pyrrolidin-1-yl)cyclohexanamine (91 mg, 0.5 mmol), 4-(trifluoromethyl)phenyl acetic acid (104 mg, 0.5 mmol), triethylamine (0.2 mL) and PyBrop (312 mg, 0.6 mmol) in CH_2Cl_2 (anhydrous, 1 mL). The crude residue was purified by column chromatography eluting with CH_2Cl_2 : MeOH : NH_3 95:5:0.1 to afford **25** (97 mg, 53 %). (^{1}H NMR, d^6 DMSO), δ = 8.82 (bs, 1H), 7.68 (d, 2H, *J* = 8.0 Hz, ArH), 7.44 (d, 2H, *J* = 8.0 Hz, ArH), 4.54 (bs, 1H), 3.85 (d, 1H, *J* = 16.3 Hz, CH_2), 3.81 (d, 1H, J = 16.3 Hz, CH_2), 3.62 (t, 1H, *J* = 11.5 Hz, CH_2), 3.38-3.35 (m, 1H), 3.29-3.21 (m, 2H), 3.19-3.15 (m, 1H), 2.90 (s, 3H, CH_3), 2.07 (d, 1H, *J* = 11.0 Hz), 1.91-1.84 (m, 3H), 1.77 (d, 1H, *J* = 12.1 Hz), 1.68 (d, 1H, *J* = 12.1 Hz), 1.57-1.55 (m, 2H), 1.53-1.50 (m, 1H), 1.31 (bs, 1H), 1.29-1.28 (m, 1H). LCMS: *m/z* 369 [M+H]$^+$, T_R = 3.1-3.2 min. HRMS (ESI) calculated for $C_{20}H_{28}F_3N_2O$ 369.2148 [M+H]$^+$found 369.2131.

***N*-Methyl-2-(naphthalen-1-yl)-*N*-(*trans*-(±))-(2-(pyrrolidin-1-yl)cyclohexyl)acetamide.HCl 26.**

Prepared according to method A from *trans*-(±)-*N*-methyl-2-(pyrrolidin-1-yl)cyclohexanamine (100 mg, 0.55 mmol), 2-(naphthalen-1-yl)acetic acid (205 mg, 1.1 mmol), HOBt (149 mg, 1.1 mmol), DIPEA (192 μL, 1.1 mmol) and EDCI (211 mg, 1.1 mmol) in DMF (anhydrous, 5 mL) in 29 % yield. ^{1}H NMR (*d6*-DMSO) δ 9.41 (br s, 1H, HCl), 8.03-8.06 (m, 1H, ArH), 7.91-7.94 (m, 1H, ArH), 7.83 (d, 1H, ArH, *J* = 8.3 Hz), 7.47-7.53 (m, 2H, ArH), 7.45 (t, 1H, ArH, *J* = 7.6 Hz), 7.35 (d, 1H, ArH, *J* = 7.1 Hz), 4.52-4.61 (br s, 1H), 4.29 (d, 1H, *J* = 16.7 Hz), 4.16 (d, 1H, *J*-16.7 Hz), 3.62-3.70 (m, 1H), 3.43-3.50 (m, 1H), 3.26-3.30 (m, 1H), 3.13-3.20 (m, 2H), 3.06 (s, 3H, CH_3), 2.07-2.13 (m, 1H), 1.94-2.01 (m, 1H), 1.85-1.92 (m, 3H), 1.75-1.81 (m, 1H), 1.69-1.75 (m, 1H), 1.61-1.67 (m, 2H), 1.46-1.56 (m, 1H), 1.23-1.33 (m, 2H). LCMS *m/z*: 351 [M+H]$^+$, T_R: 3.5 min. HRMS (ESI) calcd. for $C_{23}H_{31}N_2O$ 351.2436 [M+H]$^+$, found 351.2443.

***trans*(±)-*N*-(2-([1,1'-Biphenyl]-4-yl)ethyl)-*N*-methyl-2-(pyrrolidin-1-yl)cyclohexanamine 28**

Prepared according to method C from *trans*-(±)-*N*-methyl-2-(pyrrolidin-1-yl)cyclohexanamine (91 mg, 0.5 mmol), 4-biphenyl acetic acid (106 mg, 0.5 mmol), triethylamine (0.2 mL) and PyBrop (312 mg, 0.6 mmol) in CH_2Cl_2 (anhydrous, 1 mL). The crude residue was purified by column chromatography eluting with CH_2Cl_2 : MeOH:NH_3 95:5:0.1 to afford **28** (96 mg, 51 %). (^{1}H NMR, d^6 DMSO), δ 7.58-7.53 (m, 4H, ArH), 7.42-7.40 (m, 2H, ArH), 7.34-7.31 (m, 3H, ArH), 4.02 (d, 1H, *J* = 16.3 Hz), 3.81 (d, 1H, *J* = 16.3 Hz), 3.52 (bs, NH), 3.48 (dd, 2H, *J* = 14.1 and 7.1 Hz), 2.99 (s, 3H, Me). LCMS: *m/z* 377 $[M+H]^+$, T_R = 3.4-3.5 min. HRMS (ESI) calculated for $C_{25}H_{33}N_2O$ 377.2587 $[M+H]^+$found 377.2591.

2-(3-Bromophenyl)-*N*-methyl-*N*-((*trans*(±)-2-(pyrrolidin-1-yl)cyclohexyl)acetamide 29

Prepared according to method C from *trans*-(±)-*N*-ethyl-2-(pyrrolidin-1-yl)cyclopentanamine (130 mg, 0.5 mmol), 3-bromophenylacetic acid (108 mg, 0.5 mmol), triethylamine (0.2 mL) and PyBrop (312 mg, 0.6 mmol) in CH_2Cl_2 (anhydrous, 1 mL). The crude residue was purified by column chromatography eluting with CH_2Cl_2 : MeOH:NH_3 95:5:0.1 to afford **29** (122 mg, 64 %). (^{1}H NMR, DMSO) δ 8.82 (bs, 1H), 7.45 (s, 2H, ArH), 7.29 (t, 1H, *J* = 8.4 Hz), 7.22 (d, 1H, *J* = 7.4 Hz), 4.54 (bs, 1H), 3.79 (d, 1H, *J* = 16.1 Hz), 3.69 (d, 1H, *J* = 16.1 Hz), 3.60 (m, 1H), 3.29-3.22 (m, 2H), 3.20-3.16 (m, 1H), 2.89 (s, 3H, CH_3), 2.08 (d, 1H, *J* = 10.9 Hz), 1.91-1.83 (m, 4H), 1.78 (d, 1H, *J* = 13.0 Hz), 1.69 (d, 1H, *J* = 12.1 Hz), 1.59-1.51 (m, 3H), 1.30-1.27 (m, 1H), 1.19-1.16 (m, 1H). ^{13}C (125 MHz, [D_6] DMSO): δ = 175.9, 143.8, 128.70, 126.33, 125.06, 59.6, 58.8, 50.6, 49.7, 35.0, 30.5, 27.9, 25.7, 24.5, 23.9, 23.5, 22.9, 22.5. LCMS: *m/z* 381 and 379, ^{81}Br and ^{79}Br $[M+H]^+$ T_R = 1.1 – 1.3 min. HRMS (ESI) calculated for $C_{19}H_{28}BrN_2O$ ^{79}Br $[M+H]^+$ 379.1380 found 379.1378.

2-(3-Bromo-4-methoxyphenyl)-*N*-methyl-*N*-((*trans*(±)-2-(pyrrolidin-1-yl)cyclohexyl)acetamide 30 Prepared according to method C from *trans*-(±)-*N*-ethyl-2-(pyrrolidin-1-yl)cyclopentanamine (130 mg, 0.5 mmol), 3-bromo-4-methoxyphenylacetic acid (123 mg, 0.5 mmol), triethylamine (0.2 mL) and PyBrop (312 mg, 0.6 mmol) in CH_2Cl_2 (anhydrous, 1 mL). The crude residue was purified by column chromatography eluting with CH_2Cl_2 : MeOH : NH_3 95:5:0.1 to afford **30** (126 mg, 62 %). (^{1}H NMR, DMSO) δ 8.81 (bs, 1H), 7.45 (d, 1H, *J* = 2.1 Hz), 7.18 (dd, 1H, *J* = 8.5 and 2.1 Hz), 7.06 (d, 1H, *J* = 8.5 Hz), 4.53 (bs, 1H), 3.83 (s, 3H, CH_3), 3.71 (d, 1H, *J* = 16.2 Hz, CH_2), 3.66 (d, 1H, *J* = 16.2 Hz, CH_2), 3.62-3.57 (m, 1H), 3.39-3.34 (m, 2H), 3.29-3.19 (m, 3H), 2.89 (s, 3H, CH_3), 2.08 (d, 1H, *J* = 9.5 Hz, CH_2), 1.89-1.86 (m, 3H), 1.78 (d, 1H, *J* = 12.0 Hz, CH_2), 1.69 (d, 1H, *J* = 12.0 Hz), 1.57-1.48 (m, 2H), 1.33-1.23 (m, 3H). LCMS: *m/z* 411 and 409, ^{81}Br and ^{79}Br $[M+H]^+$, T_R = 3.2-3.4 min HRMS (ESI) calculated for $C_{20}H_{30}BrN_2O_2$ ^{79}Br $[M+H]^+$ 409.1485 found 409.1488.

2-Cyclohexyl-*N*-methyl-*N*-((*trans*(±)-2-(pyrrolidin-1-yl)cyclohexylacetamide 31

Prepared according to method C from *trans*-(±)-*N*-ethyl-2-(pyrrolidin-1-yl)cyclopentanamine (130 mg, 0.5 mmol), cyclohexaneacetic acid (71 mg, 0.5 mmol), triethylamine (0.2 mL) and PyBrop (312 mg, 0.6 mmol) in CH_2Cl_2 (anhydrous, 1 mL). The crude residue was purified by column chromatography eluting with CH_2Cl_2 :MeOH:NH_3 95:5:0.1 to afford **31** (85 mg, 55 %). (^{1}H NMR, DMSO) δ 8.78 (bs, 1H), 4.67 (bs, 1H, CH), 3.69-3.62 (t, 1H, *J* = 9.6 Hz), 3.40-3.39 (m, 1H), 3.36-3.29 (m, 2H), 3.26-3.23 (m, 1H), 2.90 (s, 3H, CH_3), 2.32 (dd, 1H, *J* = 15.7 and 6.8 Hz, CH_2), 2.25

(dd, 1H, *J* = 15.7 and 6.1 Hz, CH_2), 2.14 (d, 1H, *J* = 10.7 Hz, CH_2), 1.99-1.92 (m, 3H), 1.87-1.84 (m, 2H), 1.80-1.68 (m, 8H), 1.61 (t, 2H, *J* = 12.8 Hz, CH_2), 1.43-1.26 (m, 4H), 1.24-1.18 (m, 1H), 1.06-0.98 (m, 2H). LCMS: *m/z* 307 $[M+H]^+$, T_R = 3.2-3.3 min. HRMS (ESI) calculated for $C_{19}H_{35}N_2O$ $[M+H]^+$ 307.2744 found 307.2731.

2-(3,4-Dichlorophenyl)-*N*-methyl-*N*-(*trans*-(±))-2-(piperidin-1-yl)cyclohexyl)acetamide.HCl 33

Prepared according to method A from *trans*-(±)-*N*-methyl-2-(piperidin-1-yl)cyclohexanamine (198 mg, 0.5 mmol), 3,4-dichlorophenylacetic acid (205 mg, 1.0 mmol), HOBt (135 mg, 1.0 mmol), DIPEA (174 μL, 1.0 mmol) and EDCI (192 mg, 1.0 mmol) in DMF (anhydrous, 5 mL) in 58 % yield. ^1H NMR ($CDCl_3$) δ 10.42 (br s, 1H, HCl), 7.40 (d, 1H, ArH, *J* = 2.0 Hz), 7.37 (d, 1H, ArH, *J* = 8.2 Hz), 7.22 (dd, 1H, ArH, *J* = 8.2 and 2.0 Hz), 4.82-4.90 (m, 1H), 4.29 (d, 1H, *J* = 16.2 Hz), 3.97-3.03 (m, 1H), 3.64 (d, 1H, *J* = 16.2 Hz), 3.26-3.31 (m, 1H), 3.06-3.16 (m, 2H), 3.11 (s, 3H, CH_3), 2.94-3.02 (m, 1H), 2.51-2.60 (m, 1H), 2.23-2.33 (m, 2H), 1.88-1.98 (m, 2H), 1.80-1.87 (m, 2H), 1.70-1.78 (m, 2H), 1.57-1.66 (m, 1H), 1.45-1.54 (m, 1H), 1.21-1.40 (m, 3H). LCMS *m/z*: 383 $[M+H]^+$, T_R: 4.2 min. HRMS (ESI) calcd. for $C_{20}H_{29}N_2OCl_2$ 383.1657 $[M+H]^+$, found 383.1638.

***N*-(*trans*-(±))-2-(Azepan-1-yl)cyclohexyl)-2-(3,4-dichlorophenyl)-*N*-methylacetamide.HCl 34**

Prepared according to method A from *trans*-(±)-2-(Azepan-1-yl)-*N*-methylcyclohexanamine (105 mg, 0.5 mmol), 3,4-dichlorophenylacetic acid (205 mg, 1.0 mmol), HOBt (135 mg, 1.0 mmol), DIPEA (174 μL, 1.0 mmol) and EDCI (192 mg, 1.0 mmol) in DMF (anhydrous, 5 mL) in 67 % yield. ^1H NMR ($CDCl_3$) δ 10.57 (br s, 1H, HCl), 7.40 (d, 1H, ArH, *J* = 2.0 Hz), 7.36 (d, 1H, ArH, *J* = 8.2 Hz), 7.22 (dd, 1H, ArH, *J* = 8.2 and 2.0 Hz), 4.80-4.93 (m, 1H), 4.27-4.38 (m, 1H), 3.84-3.92 (m, 1H), 3.62-3.73 (m, 2H), 3.09 (s, 3H, CH_3), 3.04-3.11 (m, 2H), 2.95-3.02 (m, 1H), 2.38-2.46 (m, 1H), 2.18-2.29 (m, 2H), 1.92-1.99 (m, 1H), 1.70-1.88 (m, 7H), 1.51-1.61 (m, 2H), 1.42-1.51 (m, 1H), 1.32-1.42 (m,1H), 1.22-1.32 (m, 1H). LCMS *m/z*: 397 $[M+H]^+$, T_R: 4.2 min. HRMS (ESI) calcd. for $C_{21}H_{31}N_2OCl_2$ 397.1813 $[M+H]^+$, found 397.1800.

2-(3,4-Dichlorophenyl)-*N*-methyl-*N*-(*trans*-(±))-2-(4-methylpiperazin-1-yl)cyclohexyl)-acetamide.2HCl 36

Prepared according to method A from *trans*-(±)-*N*-methyl-2-(4-methylpiperazin-1-yl)cyclohexanamine (106 mg, 0.5 mmol), 3,4-dichlorophenylacetic acid (205 mg, 1.0 mmol), HOBt (135 mg, 1.0 mmol), DIPEA (174 μL, 1.0 mmol) and EDCI (192 mg, 1.0 mmol) in DMF (anhydrous, 5 mL) in 38 % yield**.** ^1H NMR ($CDCl_3$) δ 13.49 (br s, 1H, HCl), 11.89 (br s, 1H, HCl), 7.41 (d, 1H, ArH, *J* = 2.0 Hz), 7.38 (d, 1H, ArH, *J* = 8.2 Hz), 7.16 (dd, 1H, ArH, *J* = 8.2 and 2.0 Hz), 4.96-5.06 (m, 1H), 4.75-4.83 (m, 1H), 4.39-4.49 (m, 1H), 4.29-4.37 (m, 1H), 4.25 (d, 1H, *J* = 16.5 Hz), 3.99-4.10 (m, 2H), 3.63 (d, 1H, *J* = 16.5 Hz), 3.36-3.43 (m, 2H), 3.14-3.27 (m, 2H), 3.06 (s, 3H, CH_3), 2.87 (d, 3H, CH_3, *J* = 4.6 Hz), 2.30-2.37 (m, 1H), 2.00-2.06 (m, 1H), 1.85-1.91 (m, 2H), 1.60-1.69 (m, 2H), 1.23-1.42 (m, 2H). LCMS *m/z*: 398 $[M+H]^+$, T_R: 0.9 min. HRMS (ESI) calcd. for $C_{20}H_{30}N_3OCl_2$ 398.1766 $[M+H]^+$, found 398.1767.

2-(3,4-Dichlorophenyl)-*N*-(*trans*-(±))-2-(3,3-difluoropyrrolidin-1-yl)cyclohexyl-*N*-methylacetamide 37

Prepared according to method C from (*trans*-(±))-2-(3,3-difluoropyrrolidin-1-yl)-*N*-methylcyclohexylanamine (100 mg, 0.46 mmol), 3,4-dichlorophenyl acetic acid (94

mg, 0.5 mmol), triethylamine (0.2 mL) and PyBrop (285 mg, 0.55 mmol) in CH_2Cl_2 (anhydrous, 1 mL). The crude residue was purified by column chromatography eluting with CH_2Cl_2 :MeOH:NH_3 95:5:0.1 to afford **37** (10 mg, 6 %). (^{1}H NMR, $CDCl_3$), δ = 7.37 (d, 1H, *J* = 8.3 Hz, ArH), 7.33 (d, 1H, *J* = 2.1 Hz, ArH), 7.09 (dd, 1H, *J* = 8.3 and 2.2 Hz, ArH), 4.47 (bs, 1H), 3.65 (d, 2H, *J* = 6.8 Hz), 3.08-2.84 (m, 1H), 2.90-2.84 (m, 1H), 2.83 (s, 3H, CH_3), 2.67-2.60 (m, 2H), 2.17-2.02 (m, 2H), 1.89-1.80 (m, 2H), 1.78-1.70 (m, 2H), 1.43-1.37 (m, 1H), 1.35 (dt, 1H, *J* = 12.7, 3.2 Hz), 1.25-1.21 (m, 1H), 1.13 (dt, 1H, *J* = 12.7 and 3.2 Hz). LCMS: *m/z* 405 $[M+H]^+$, T_R = 3.6-3.7 min.

2-(3,4-Dichlorophenyl)-N-ethyl-N-(trans(±)-2-(pyrroldin-1-yl)cyclopentyl)acetamide 39

Prepared according to method C from *trans*-(±)-*N*-ethyl-2-(pyrrolidin-1-yl)cyclopentanamine (130 mg, 0.5 mmol), 3,4-dichlorophenyl acetic acid (130 mg, 0.5 mmol), triethylamine (0.2 mL) and PyBrop (312 mg, 0.6 mmol) in CH_2Cl_2 (anhydrous, 1 mL). The crude residue was purified by column chromatography eluting with CH_2Cl_2:MeOH:NH_3 95:5:0.1 to afford **39** (123 mg, 64 %). (^{1}H NMR, $CDCl_3$), δ 7.59-7.54 (m, 1H, ArH), 7.53-7.52 (m, 1H, ArH), 7.24 (dd, 1H, ArH, *J* = 8.1, 2.0 Hz), 3.81-3.76 (m, 1H), 3.68-3.59 (m, 1H), 3.31-3.26 (m, 1H), 3.05-3.00 (m, 1H), 2.41-2.37 (m, 1H), 1.88-1.82 (m, 1H), 1.71-1.67 (m, 2H), 1.58 (bs, 3H), 1.51-1.47 (m, 4H), 1.19 (bs, 2H), 1.16 (t, 4H, *J* = 7.0 Hz), 1.03 (t, 1H, *J* = 7.0 Hz). LCMS: *m/z* 383 $[M+H]^+$, T_R = 3.1-3.3 min. HRMS (ESI) calculated for $C_{20}H_{29}Cl_2N_2O$ ^{35}Cl $[M+H]^+$ 383.1660 found 383.1651.

***trans*(±)-*N*-(*tert*-Butyl)-2-(3,4-dichlorophenyl)-*N*-((trans(±)-2-(pyrrolidin-1-yl)cyclohexyl)acetamide 40**

Prepared according to method C from *trans*-(±)--*N*-(*tert*-butyl)-2-(pyrrolidin-1-yl)cyclohexanamine (130 mg, 0.64 mmol), 3,4-dichlorophenyl acetic acid (131 mg, 0.64 mmol), triethylamine (0.26 mL) and PyBrop (399 mg, 0.77 mmol) in CH_2Cl_2 (anhydrous, 1.5 mL). The crude residue was purified by column chromatography eluting with CH_2Cl_2 : MeOH : NH_3 95:5:0.1 to afford **40** (173 mg, 66 %). (^{1}H NMR, $CDCl_3$), δ 7.51 (d, 2H, *J* = 8.3 Hz), 7.29 (dd, 1H, *J* = 8.3 and 2.0 Hz, ArH), 4.01 (bs, 2H), 3.43 (bs, 1H), 2.91-2.86 (m, 1H), 2.72-2.70 (m, 2H), 2.66-2.62 (m, 2H), 2.01 (bs, 1H), 1.92 (bs, 1H), 1.85-1.83 (m, 2H), 1.77-1.74 (m, 1H), 1.71 (s, 8H, *t*-butyl), 1.35 (bs, 1H), 1.11 (bs, 1H), 1.04-0.99 (m, 2H), 0.91-0.88 (m, 1H). LCMS: *m/z* 395 and 397, ^{35}Cl and ^{37}Cl $[M+H]^+$, T_R = 3.2-3.4 min. HRMS (ESI) calculated for $C_{21}H_{29}Cl_2N_2O$ ^{35}Cl $[M+H]^+$ 395.1651 found 395.1654.

***trans*(±)-*N*-Benzyl-2-(3,4-dichlorophenyl)-*N*-((trans(±)-2-(pyrrolidin-1-yl)cyclohexyl)acetamide 41**

Prepared according to method C from *trans*-(±)-*N*-benzyl-2-(pyrrolidin-1-yl)cyclohexanamine (198 mg, 0.75 mmol), 3,4-dichlorophenyl acetic acid (153 mg, 0.75 mmol), triethylamine (0.30 mL) and PyBrop (478 mg, 0.90 mmol) in CH_2Cl_2 (anhydrous, 1.5 mL). The crude residue was purified by column chromatography eluting with CH_2Cl_2:MeOH:NH_3 95:5:0.1 to afford **41** (47 mg, 14 %). (^{1}H NMR, DMSO), δ = 9.97 (bs, 1H), 7.51 (d, 1H, *J* = 8.3 Hz, ArH), 7.42 (d, 1H, *J* = 5.9 Hz), 7.39 (d, 2H, *J* = 7.2 Hz, ArH), 7.31 (d, 3H, *J* = 7.6 Hz), 7.17 (d, 1H, *J* = 7.9 Hz), 5.02-4.98 (m, 1H), 4.71 (d, 1H, *J* = 18.3 Hz), 4.17 (bs, 1H), 3.80 (bs, 1H), 3.30-3.24 (m, 2H), 3.20-3.15 (m, 1H), 2.07 (d, 1H, *J* = 10.9 Hz), 1.98-1.95 (m, 1H), 1.90-1.85 (m, 4H), 1.73-1.65 (m, 2H), 1.61-1.57 (m, 1H), 1.52 (d, 1H, *J* = 11.3Hz), 1.25-1.17 (m,

2H). LCMS: *m/z* 445 and 447, ^{35}Cl and ^{37}Cl $[M+H]^+$, T_R = 3.5-3.6 min. HRMS (ESI) calculated for $C_{25}H_{31}Cl_2N_2O$ ^{35}Cl $[M+H]^+$ 445.1808 found 445.1802.

2-(3,4-Dichlorophenyl)-*N*-methyl-*N*-(*trans*(±)-4-(pyrrolidin-1-yl)tetrahydrofuran-3-yl)acetamide 42

Prepared according to method C from *trans*-(±)-*N*-methyl-4-(pyrrolidin-1-yl)tetrahydrofuran-3-amine (91 mg, 0.5 mmol), 3,4-dichlorophenyl acetic acid (102 mg, 0.5 mmol), triethylamine (0.2 mL) and PyBrop (312 mg, 0.6 mmol) in CH_2Cl_2 (anhydrous, 1 mL). The crude residue was purified by column chromatography eluting with CH_2Cl_2:MeOH:NH3 95:5:0.1 to afford **42** (38 mg, 21 %). (^{1}H NMR, $CDCl_3$), δ 7.41 (d, 1H, ArH, *J* = 1.8 Hz), 7.39 (dd, 1H, ArH, *J* = 3.4, 1.8 Hz), 7.15-7.12 (m, 1H, ArH), 2.93 (bs, 1H, CH), 2.87 (bs, 1H, CH), 2.53 (bs, 3H, CH_3), 2.00-1.93 (m, 2H, CH_2), 1.76-1.73 (m, 4 H, CH_2), 1.69-1.58 (m, 8H, CH_2). LCMS: *m/z* 357 and 359 ^{35}Cl and ^{37}Cl $[M+H]^+$, T_R = 3.6-3.7 min. HRMS (ESI) calculated for $C_{17}H_{23}Cl_2N_2O_2$ 357.1131 ^{35}Cl $[M+H]^+$found 357.1145.

2-(3,4-Dichlorophenyl)-*N*-methyl-*N*-(*trans*(±)-2-(pyrrolidin-1-yl)cyclopentyl)acetamide 43

Prepared according to method C from *trans*-(±)-*N*-methyl-2-(pyrrolidin-1-yl)cyclopentanamine (134 mg, 0.8 mmol), 3,4-dichlorophenyl acetic acid (164 mg, 0.8 mmol), triethylamine (0.32 mL) and PyBrop (499 mg, 0.96 mmol) in CH_2Cl_2 (anhydrous, 2 mL). The crude residue was purified by column chromatography eluting with CH_2Cl_2 : MeOH : NH_3 95:5:0.1 to afford **43** (37 mg, 13 %). ^{1}H NMR, d6 DMSO, δ 7.59 (d, 1H, *J* = 8.3 Hz), 7.51 (d, 1H, *J* = 2.0 Hz), 7.23 (dd, 1H, *J* = 8.3 and 2.0 Hz), 5.24 (bs, 1H), 4.19 (dd, 1H, *J* = 10.0, 7.6 Hz), 3.98 (bs, 1H), 3.96 (dd, *J* = 16.8 and 7.6 Hz), 3.86 (dd, 1H, *J* = 10.3, 6.3), 3.78 (d, 2H, *J* = 3.3 Hz), 3.74 (dd, 1H, *J* = 9.2 and 5.5 Hz), 3.50 (bs, 2H), 2.96 (bs, 2H), 1.92 (bs, 2H), 1.87 (bs, 2H). LCMS: *m/z* 355 and 357 ^{35}Cl and ^{37}Cl $[M+H]^+$, T_R = 3.6-3.7 min. HRMS (ESI) calculated for $C_{18}H_{25}Cl_2N_2O$ 355.1338 ^{35}Cl $[M+H]^+$found 355.1334.

2-(3,4-Dichlorophenyl)-*N*-(2-(pyrrolidin-1-yl)ethyl)acetamide 45

Prepared according to method A from 1-(2-Aminoethyl)-pyrrolidine (250 μL, 1.99 mmol), 3,4-dichlorophenylacetic acid (814 mg, 4.0 mmol), HOBt (540 mg, 4.0 mmol), DIPEA (384 μL, 4.0 mmol) and EDCI (768 mg, 4.0 mmol) in DMF (anhydrous, 10 mL) in 70% yield**.** ^{1}H NMR ($CDCl_3$) δ 7.38-7.41 (m, 2H, ArH), 7.13 (dd, 1H, ArH, *J* = 8.2 and 2.1 Hz), 6.07-6.19 (br s, 1H, NH), 3.49 (s, 2H), 3.32 (q, 2H, *J* = 5.9 Hz), 2.55 (t, 2H, *J* = 5.9 Hz), 2.42-2.47 (m, 4H), 1.72-1.75 (m, 4H). LCMS *m/z*: 301 $[M+H]^+$, T_R: 4.3 min. HRMS (ESI) calcd. for $C_{14}H_{19}N_2OCl_2$ 301.0874 $[M+H]^+$, found 301.0860.

***N*-Methyl-*N*-((*trans*(±)-2-(pyrrolidin-1-yl)cyclohexyl)-[1,1'-biphenyl]-3-carboxamide 49**

Prepared according to method C from *trans*-(±)-*N*-ethyl-2-(pyrrolidin-1-yl)cyclopentanamine (130 mg, 0.5 mmol), 3-biphenylcarboxylic acid (99 mg, 0.5 mmol), triethylamine (0.2 mL) and PyBrop (312 mg, 0.6 mmol) in CH_2Cl_2 (anhydrous, 1 mL). The crude residue was purified by column chromatography eluting with CH_2Cl_2 : MeOH : NH_3 95:5:0.1 to afford **49** (120 mg, 66 %). (^{1}H NMR, DMSO), δ 8.91 (bs, 1H), 7.78 (d, 1H, *J* = 7.4 Hz, PhH), 7.73 (s, 1H), 7.71 (d, 2H, *J* = 7.1 Hz, PhH), 7.57 (t, 1H, *J* = 7.3 Hz, PhH), 7.51 (t, 3H, *J* = 7.5 Hz, PhH), 7.42 (t, 1H, *J* = 7.4 Hz, PhH), 4.69 (bs, 1H, 3.74 (bs, 1H), 3.31 (s, 3H, CH_3), 3.04-3.01 (m, 2H), 2.85 (bs,

2H), 2.14 (bs, 1H), 1.94-1.88 (m, 5H), 1.75-1.73 (m, 2H), 1.71-1.69 (m, 1H), 1.65-1.63 (m, 1H), 1.62-1.60 (m, 1H). LCMS: *m/z* 363 $[M+H]^+$, T_R = 3.2-3.3 min. HRMS (ESI) calculated for $C_{24}H_{31}N_2O$ 363.2431 $[M+H]^+$found 363.2418.

_N_-Methyl-_N_-((_trans_(±)-2-(pyrrolidin-1-yl)cyclohexyl)-[1,1'-biphenyl]-4-carboxamide 50

Prepared according to method C from *trans*-(±)-*N*-ethyl-2-(pyrrolidin-1-yl)cyclopentanamine (130 mg, 0.5 mmol), 4-biphenylcarboxylic acid (99 mg, 0.5 mmol), triethylamine (0.2 mL) and PyBrop (312 mg, 0.6 mmol) in CH_2Cl_2 (anhydrous, 1 mL). The crude residue was purified by column chromatography eluting with CH_2Cl_2 : MeOH : NH_3 95:5:0.1 to afford **50** (20 mg, 11 %). (^{1}H NMR, $CDCl_3$), δ = 7.68 (d, 2H, *J* = 8.2 Hz, ArH), 7.62 (d, 2H, *J* = 7.2 Hz, ArH), 7.57 (bd, 2H, *J* = 8.2 Hz, ArH), 7.48 (t, 2H, *J* = 7.2 Hz, PhH), 7.40 (tt, 1H, *J* = 7.4 and 1.2 Hz), 4.65 (bs, 1H), 3.80-3.72 (bs, 2H), 3.42-3.27 (m, 2H), 3.01 (s, 3H, CH_3), 2.25 (bs, 1H, *J* = 12 Hz), 2.08 (bs, 4H), 2.00-1.89 (m, 3H), 1.88-1.82 (m, 1H), 1.66-1.58 (bs, 3H, 1.50-1.43 (m, 1H). LCMS: *m/z* 363 $[M+H]^+$, T_R = 2.8-3.0 min.

2-(3,4-Dichlorophenoxy)-_N_-methyl-_N_-(_trans_-(±))-2-(pyrrolidin-1-yl)cyclohexyl)acetamide.HCl 51

Prepared according to method A from *trans*-(±)-*N*-Methyl-2-(pyrrolidin-1-yl)cyclohexanamine (100 mg, 0.55 mmol), 2-(3,4-Dichlorophenoxy)acetic acid (243 mg, 1.1 mmol), HOBt (149 mg, 1.1 mmol), DIPEA (192 μL, 1.1 mmol) and EDCI (211 mg, 1.1 mmol) in DMF (anhydrous, 5 mL) in 40 % yield. ^{1}H NMR (*d6*-DMSO) δ 9.75 (br s, 1H, HCl), 7.47 (d, 1H, ArH, *J* = 8.9 Hz), 7.39 (d, 1H, ArH, *J* = 2.9 Hz), 7.05 (dd, 1H, ArH, *J* = 8.9 and 2.9 Hz), 5.09 (d, 1H, *J* = 16.0 Hz), 4.93 (d, 1H, *J* = 16.0 Hz), 4.43-4.54 (m, 1H), 4.57-4.65 (m, 1H), 3.40-3.49 (m, 1H), 3.23-3.31 (m, 1H), 3.14-3.23 (m, 1H), 3.05-3.13 (m, 1H), 2.90 (s, 3H, CH_3), 2.02-2.09 (m, 1H), 1.92-1.99 (m, 1H), 1.82-1.91 (m, 3H), 1.74-1.80 (m, 1H), 1.67-1.73 (m, 1H), 1.56-1.63 (m, 2H), 1.47-1.56 (m, 1H), 1.20-1.37 (m, 2H). LCMS *m/z*: 385 $[M+H]^+$, T_R: 3.8 min. HRMS (ESI) calcd. for $C_{19}H_{27}N_2O_2Cl_2$ 385.1450 $[M+H]^+$, found 385.1443.

3-(4-Chlorophenyl)-_N_-methyl-_N_-(_trans_-(±))-2-(pyrrolidin-1-yl)cyclohexyl)propanamide.HCl 52

Prepared according to method A from *trans*-(±)-*N*-Methyl-2-(pyrrolidin-1-yl)cyclohexanamine (100 mg, 0.55 mmol), 3-(4-Chlorophenyl)propanoic acid (203 mg, 1.1 mmol), HOBt (149 mg, 1.1 mmol), DIPEA (192 μL, 1.1 mmol) and EDCI (211 mg, 1.1 mmol) in DMF (anhydrous, 5 mL) in 41 % yield. ^{1}H NMR (*d6*-DMSO) δ 9.69 (br s, 1H, HCl), 7.27-7.33 (m, 4H, ArH), 4.50-4.61 (m, 1H), 3.49-3.58 (m, 1H), 3.30-3.44 (m, 2H), 3.09-3.25 (m, 3H), 2.85 (s, 3H, CH_3), 2.78-2.85 (m, 2H), 2.55-2.65 (m, 1H), 2.03-2.09 (m, 1H), 1.86-1.94 (m, 1H), 1.58-1.86 (m, 3H), 1.72-1.78 (m, 1H), 1.65-1.71 (m, 1H), 1.46-1.57 (m, 3H), 1.18-1.36 (m, 2H). LCMS *m/z*: 349 $[M+H]^+$, T_R: 3.8 min. HRMS (ESI) calcd. for $C_{20}H_{30}N_2OCl$ 349.2047 $[M+H]^+$, found 349.2040.

_N_-Methyl-4-phenyl-_N_-((_trans_(±))-2-(pyrrolidin-1-yl)cyclohexyl)butanamide 53

Prepared according to method C from *trans*-(±)-*N*-ethyl-2-(pyrrolidin-1-yl)cyclopentanamine (130 mg, 0.5 mmol), 4-phenylbutyric acid (82 mg, 0.5 mmol), triethylamine (0.2 mL) and PyBrop (312 mg, 0.6 mmol) in CH_2Cl_2 (anhydrous, 1 mL). The crude residue was purified by column chromatography eluting with CH_2Cl_2 : MeOH : NH_3 95:5:0.1 to afford **53** (37 mg, 23 %). (^{1}H NMR, DMSO), δ = 8.61 (bs, 1H), 7.22 (t, 2H, *J* = 7.4 Hz, PhH), 7.14 (d, 2H, *J* = 6.9 Hz), 7.11 (d, 1H, *J* = 7.3 Hz),

4.48 (bs, 1H), 3.48 (bs, 1H), 3.23 (s, 3H, CH_3), 3.21-3.16 (m, 2H), 3.08-3.07 (m, 1H), 2.71 (s, 2H), 2.57-2.51 (m, 2H), 2.30-2.25 (m, 2H), 1.99 (d, 1H, *J* = 9.4 Hz), 1.82-1.69 (m, 7H), 1.62 (d, 1H, *J* = 13 Hz), 1.48-1.41 (m, 2H), 1.25-1.20 (m, 2H), 1.17-1.15 (m, 1H). LCMS: *m/z* 329 $[M+H]^+$, T_R = 3.0-3.1 min. HRMS (ESI) calculated for $C_{21}H_{33}N_2O$ 329.2587 $[M+H]^+$found 329.2573.
